# Biofilm formation during pneumococcal carriage imprints naturally acquired humoral immunity

**DOI:** 10.1371/journal.ppat.1013826

**Published:** 2026-07-28

**Authors:** Jessica R. Lane, Henry Mauser, Silvia E. Santana-Krimskaya, Vahini S. Konda, Andrew DePass, Giuseppe Ercoli, Federico I. Prokopczuk, Mohammed Mohasin, Adonis D’Mello, Hervé Tettelin, Jeremy S. Brown, Luis F. Reyes, Carlos J. Orihuela

**Affiliations:** 1 Department of Microbiology, Heersink School of Medicine, The University of Alabama at Birmingham, Birmingham, Alabama, United States of America; 2 Centre for Inflammation and Tissue Repair, Division of Medicine, University College of London, London, United Kingdom; 3 Institute for Genome Sciences, Department of Microbiology & Immunology, University of Maryland School of Medicine, Baltimore, Maryland, United States of America; 4 Unisabana Center for Translational Science, Universidad de La Sabana, Chia, Colombia; 5 ISARIC, Pandemic Sciences Institute, Oxford University, Oxford, United Kingdom; Tufts Univ School of Medicine, UNITED STATES OF AMERICA

## Abstract

*Streptococcus pneumoniae* (*Spn*) colonization of the nasopharynx is a prerequisite for transmission and invasive disease. To investigate how repeated asymptomatic colonization shapes immunity and influences bacterial traits, we developed the Repeated Asymptomatic Murine Pneumococcal Colonization (RAMPC_3_) model using strains belonging to serotypes: 2 (D39), 3 (WU2), and 4 (TIGR4). Sequential colonization revealed strain- and exposure-order-dependent effects on bacterial burden, with initial colonization yielding robust carriage and subsequent exposures resulting in diminished burden and rapid clearance. Humoral profiling demonstrated antigenic imprinting: the first colonizing strain largely determined IgG and IgA specificity against bacterial proteins, with minimal diversification or expansion after repeated exposures. Reactivity was strongest for biofilm-associated antigens correlating with each strain’s biofilm-forming capacity. Notably, experiments using human sera from naturally colonized adults mirrored these findings, with reactivity favoring biofilm antigens independent from capsule. Partial protection as result of colonization was demonstrated as triple-colonized mice had reduced mortality following pneumococcal pneumonia challenge. Likewise, mice colonized with biofilm deficient versions of TIGR4 and then challenged intratracheally with a serotype 6A (6A-10) strain were more likely to develop bacteremia, underscoring the contribution of the biofilm-associated host response to immunity. Finally, IgA responses in nasal-associated lymphoid tissue paralleled serum IgA patterns, validating systemic measurements as a proxy for mucosal immunity. These results reveal that biofilm formation during colonization is a key determinant of humoral immunity and contributes to systemic protection, providing insight into pneumococcal biology and informing strategies to design next-generation interventions.

## Author summary

*Streptococcus pneumoniae* (*Spn*) is a leading cause of pneumonia, meningitis, and sepsis, yet its primary lifestyle is asymptomatic colonization of the nasopharynx. Understanding how colonization shapes immunity and bacterial physiology is critical for predicting disease risk and improving future vaccines. Using a novel murine model of repeated colonization and human sera from naturally colonized adults, we show that humoral immunity is strongly biased toward antigens expressed during biofilm growth, the predominant mode of *Spn* in the nasopharynx, rather than planktonic forms. Moreover, this response is strain-dependent with the ability of the first colonizing strain to form biofilms influencing the humoral response to subsequent exposures, i.e., imprinting, while conferring partial protection against pneumonia and development of bacteremia. Importantly, this response was capsule type independent. These findings provide fundamental insight into pneumococcal biology and the host response, suggesting that targeting biofilm-associated antigens may improve vaccine design or strategies to prevent transmission and invasive disease.

## Introduction

*Streptococcus pneumoniae* (*Spn*), commonly known as the pneumococcus, is a Gram-positive bacterium and a major cause of otitis media, community-acquired pneumonia, bacteremia, and meningitis [1]. As a pathobiont that colonizes the nasopharynx, the risk of life-threatening infection is greatest among infants and the elderly, where host immunity is diminished [2–9]. Nasopharyngeal carriage is highly prevalent among young children, particularly in daycare settings, and serves as an important immunizing event [10–12]. Accordingly, multiple studies have demonstrated the generation of antibody against protein and non-protein antigens following pneumococcal colonization including to its exopolysaccharide capsule, pneumococcal surface protein A (PspA), the pore-forming toxin pneumolysin, and many other molecules; each conferring varying degrees of protection [13–15]. Repeated exposures are thought to strengthen these responses, reducing colonization burden and conferring protection against disease [16–19].

Naturally acquired immunity to *Spn* primarily arises from the host response to its protein antigens, whereas licensed pneumococcal vaccines induce immunity by targeting the bacterium’s capsule [20]. *Spn* encompasses more than 100 biochemical and antigenically distinct serotypes [21–23], and therefore antibodies against one capsule type are unable to protect against infection caused by *Spn* carrying a different capsule type [24]. Licensed conjugate vaccines achieve broad protection by being polyvalent, currently incorporating capsular polysaccharide (CPS) from up to 21 distinct serotypes [25]. Importantly, the immunological steps by which *Spn* colonization primes protein-specific immunity remain poorly defined and are an active area of investigation [26,27]. This is a critical gap in our understanding of pneumococcal host-pathogen interactions and represents an opportunity to improve the design of prophylactic measures.

*Spn* has been shown to grow as aggregates or biofilms during nasopharyngeal colonization, which, when compared to the planktonic state, enhances adhesion to host surfaces, provides protection against host-derived antimicrobial molecules, and promotes survival on fomites [28–35]. In contrast, during pneumonia and invasive disease, pneumococci are found in a planktonic phenotype, i.e., growing as individual diplococci in suspension, a state that facilitates complement evasion [36–38]. These two growth modes are driven by environmental and physiological cues and are transcriptionally and phenotypically distinct [39–42]. This distinction is critical because most studies examining antigenic responses to *Spn* colonization have not taken into account the biofilm phenotype and instead relied primarily on planktonic cell lysates to identify immunogenic proteins [19,43–45]. Consequently, gaps exist in our understanding of how biofilm-specific antigens contribute to naturally acquired immunity and protection against *Spn*.

In this study we utilize a novel murine model of repeated asymptomatic colonization to characterize the development of humoral immunity against *Spn* and assess the contribution of antigens produced during biofilm and planktonic growth. We evaluate how repeated colonization influences bacterial burden, the duration of carriage, and in turn, protection against lethal challenge by unrelated strains of *Spn*. Using human sera, we validate the immunogenicity of biofilm-associated antigens, confirming their potential as meaningful targets. Our findings provide insight into the dynamics of the immune response to *Spn* colonization, describe a compelling outcome for early exposure, and provide important new considerations for the selection of protein antigens for a next-generation pneumococcal vaccine.

## Results

### *Spn* burden in a murine repeated asymptomatic colonization model depends on strain and exposure order

**Fig 1:**
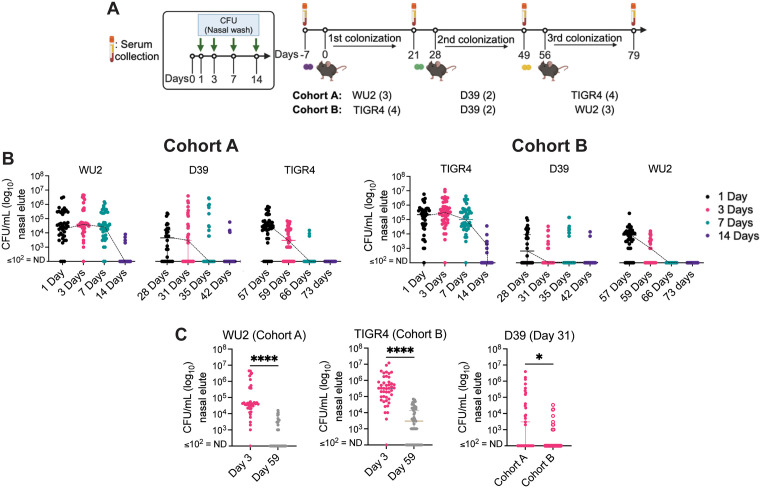
*Spn* burden in a murine repeated asymptomatic colonization model depends on strain and order exposure. **(A)** Schematic of the Repeated Asymptomatic Murine Pneumococcal Colonization (RAMPC_3_) model (see methods). 9-week-old C57BL/6J male and female mice were intranasally inoculated with 10^4^ CFU of WU2 (Cohort A) or TIGR4 (Cohort B) for the first colonization event, followed by D39 for the second, and the final and third event with TIGR4 or WU2, respectively. **(B)** Bacterial burden was determined over a 2-week period post-inoculation by colony forming units (CFUs) obtained from nasal washes with saline. **(C)** Bacterial burden for each strain at Day 3 (pink) and Day 59 (gray) or Day 31 (D39 only). Each dot is one mouse sample. N = 40-45 per group over three separate experiments. Not detected (ND) ≤ 10^2^ CFU/mL. Mann-Whitney t-test and median with 95% confidence interval (CI). * = p ≤ 0.0332; **** = p ≤ 0.0001. Panel A created in BioRender. Orihuela, C. (2026) https://BioRender.com/hfo4l9o.

To characterize how the adaptive immune response develops following repeated exposures to *Spn*, we created the Repeated Asymptomatic Murine Pneumococcal Colonization (RAMPC_3_) model using serotype 2 strain D39, serotype 3 strain WU2, and serotype 4 strain TIGR4. These strains represent distinct genetic lineages and are frequently used by *Spn* investigators [46]. RAMPC_3_ involves sequential, non-overlapping colonization of mice with these strains over a three-month period (Fig 1A). To dissect strain-level effects, two cohorts of mice were colonized with these strains but in opposite order: Cohort A (WU2 → D39 → TIGR4) and Cohort B (TIGR4 → D39 → WU2). Nasal wash (NW) bacterial burden was quantitated for each challenge strain on post-inoculation days 1, 3, 7, and 14, and serum was collected on days −7, 21, 49, and 79. Colonization by each strain did not persist beyond 28 days.

Our first observation was that initial colonization, regardless of the strain used, resulted in robust carriage that remained stable for at least seven days. For Cohort A, median bacterial titers in NW of mice colonized with WU2 exceeded 10^4^ CFU/mL for the first 7 days whereas for Cohort B, median bacterial titers in NW for TIGR4 exceeded 10^5^ CFU/mL over the same time. Second exposures, both with D39, resulted in median initial burdens that were 10-fold and 100-fold lower than for WU2 or TIGR4, respectively, with most animals having cleared D39 within 7 days of challenge (corresponding to day 31; Fig 1B). Following the third colonization, median bacterial burden for WU2 and TIGR4 in NW differed by more than 100-fold depending on whether the strain was first or third in the corresponding cohort (Fig 1C). For Cohort B, peak D39 burden in the nasopharynx (day 1 after inoculation) following TIGR4 colonization was lower than that observed in naïve mice colonized with D39 at the same time point (S1A and S1B Fig), a finding suggestive of strain effects. Overall, our results with RAMPC_3_ align with human epidemiology [16–19], i.e., repeated colonization can occur but coincides with diminished burden and more rapid clearance. This decline likely reflects the development of adaptive immunity.

### The first colonization event imprints a humoral immune response to pneumococcal proteins consistent throughout repeated colonization

**Fig 2:**
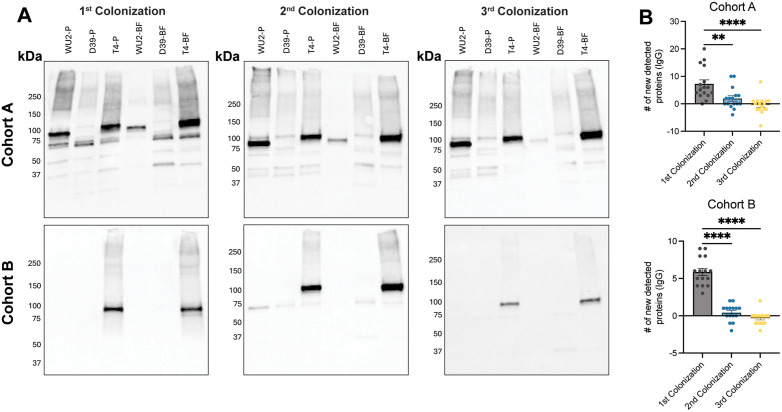
The first colonization event imprints a mucosal and systemic antibody response to the proteins that persist following repeated colonization. **(A)** Equal amounts of whole bacterial cell lysates grown planktonically (P) or in a biofilm (BF) from three *Spn* strains WU2 (serotype 3), D39 (serotype 2), and TIGR4 (serotype 4) were analyzed by immunoblot. Membranes were probed individually with mouse sera (1:1000) from Cohort A and Cohort B RAMPC_3_ mice after the first, second, and third colonization events and secondary α-mouse IgG (1:10000). Representative blots shown. **(B)** The number of new protein antigens detected by IgG on immunoblots from Cohort A and Cohort B RAMPC_3_ mice after the first, second, and third colonization events (see methods). N = 15-16 over two separate experiments. One-way ANOVA and mean with standard deviation. ** = p ≤ 0.002; **** = p ≤ 0.0001.

Using RAMPC_3_ serum samples, we examined how humoral immunity developed over time. Surprisingly, instead of increasing the diversity of antigens recognized, immunoblots using equal amounts of pneumococcal whole cell lysates (WCL) and serum samples from sequentially colonized mice revealed minimal acquisition of new IgG-reactive protein bands after the second and third colonization events, regardless of cohort (Figs 2A, 2B and S2A–S2C). Though sera from both cohorts detected on average 6–7 proteins in our WCL panel, strain-specific effects were evident: Cohort A showed a broader range of protein recognition, up to 20 bands, compared to Cohort B, up to 9 bands. A similar pattern was observed for IgA, albeit less pronounced (S3A and S3B Fig). These findings indicated the first colonizing *Spn* strain encountered largely determines the humoral immune response to pneumococcal proteins even after repeated colonization events, i.e., a version of antigenic imprinting.

**Fig 3:**
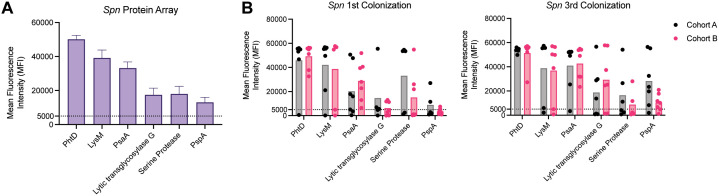
*Spn* protein array identifies specific antigens following repeated colonization. A pneumococcal protein array was constructed with 254 highly antigenic proteins. Proteins were selected from a panel of *Spn* strains and were conserved for recognition by IgG from healthy human adults (Croucher *et al.* 2017). Sera from RAMPC_3_ colonized mice after the first and third colonization events were used to probe the protein array (1:100) (see methods). **(A)** Both cohorts and timepoints combined. Each dot is one mouse sample. N = 14 over two separate experiments. **(B)** Cohorts and timepoints expanded. N = 5-7 per Cohort over two separate experiments. Limit of detection was MFI = 5000. Mean with standard deviation.

As the detected proteins are immunogenic and therefore viable targets for intervention, we furthered this line of investigation and probed a previously described *Spn* protein array using sera from both RAMPC_3_ cohorts after the first and third colonization events [47–49]. The six strongest signals included Pneumococcal histidine triad protein D (PhtD), Lysozyme M (LysM), Pneumococcal surface adhesin A (PsaA), Lytic transglycosylase G, a serine protease (Subtilase family-S8 subtilisin), and PspA (Fig 3A). These proteins play key roles in surface interactions of *Spn*, including adhesion and cell wall hydrolysis, and have been shown to be differentially expressed during colonization compared to bloodstream infection (Table 1) (S4A Fig and S2 Table). When stratified by cohort and colonization event, both cohorts of mouse sera consistently showed PhtD as the top hit followed by LysM, across all samples (Figs 3B and S4B). When analyzed, reactivity to individual proteins did not differ between the first and third colonization event, the exception being PspA which was increased following the third colonization for both Cohort A and B (S4C Fig).

**Table 1:** Antigenic pneumococcal proteins from colonized mice.

Antigen	Description	Protective (?)
Pneumococcal histidine triad protein D (PhtD) (93 kDa) (SP_1174)	Surface adhesin	Sepsis model (subcutaneous)^72^
Lysozyme M (LysM); host (17 kDa) (SP_0107)	Hydrolysis of peptidoglycan cell wall and bacterial lysis at mucosal surfaces	Literature not found
Pneumococcal surface adhesin A (PsaA) (35 kDa) (SP_1650)	Surface adhesin, lipoprotein Mn^2 +^ transporter, protects against oxidative stress	Single carriage event (enhanced when combined with PspA), not protective in sepsis model (intraperitoneal)^68–70^
Lytic transglycosylase G (61 kDa) (SP_1518)	Cell wall hydrolase	Target in combination with antibiotic treatment^73^
Serine protease (Subtilase family-S8 subtilisin) (150–240 kDa) (SP_0641a)	Surface adhesin during colonization (unknown host target)	Literature not found
Pneumococcal surface protein A (PspA) (67–99 kDa) (SP_0117)	Choline-binding protein, surface adhesin, protects against C-reactive protein	Colonization, pneumonia, and sepsis models^13, 71^

### Repeated murine *Spn* colonization elicits a strain-dependent humoral immune response associated with biofilm formation

**Fig 4:**
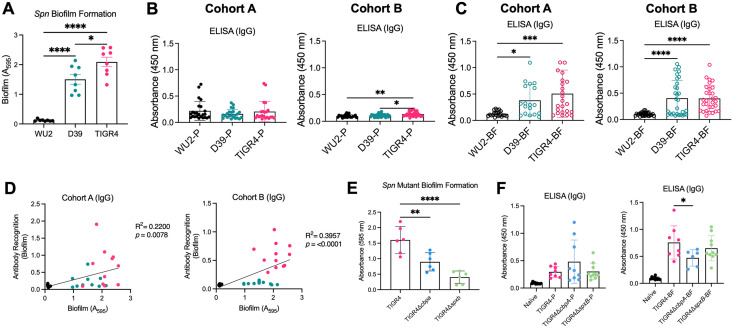
Repeated murine *Spn* colonization elicits a strain-dependent humoral response associated with biofilm formation. **(A)** *Spn* lab strain’s (WU2, D39, TIGR4) ability to form biofilms as measured by crystal violet assay (see methods). N = 8-10. Equal amounts of whole bacterial cell lysates grown **(B)** planktonically (P) or in a **(C)** biofilm (BF) from three *Spn* strains WU2 (serotype 3), D39 (serotype 2), and TIGR4 (serotype 4) were run on ELISAs and individually probed with serum (1:1000) from RAMPC_3_ mice in both Cohort A and Cohort B after the third colonization event. Secondary antibody α-mouse IgG (1:10000). Each dot is one mouse sample. N = 24-30 over two separate experiments. **(D)** Linear regression correlation between ability of *Spn* strains (strain-assigned colors corresponding to panel A) to form biofilms and a ratio of antibody recognition to biofilm antigens. Each dot is one mouse sample. N = 10-12. **(E)** Two isogenic TIGR4 mutant’s (∆*cbpA* and ∆*spxB*) ability to form biofilms as measured by crystal violet assay (see methods). N = 6 over one experiment. **(F)** Equal amounts of whole bacterial cell lysates grown planktonically (P) or in a biofilm (BF) from TIGR4 and two isogenic TIGR4 mutants were run on ELISAs and individually probed with serum (1:1000) from mice colonized once with each strain for 21 days. Secondary antibody α-mouse IgG (1:10000). Each dot is one mouse. Outliers were removed using the ROUT method. N = 6-10 over two experiments. One-way ANOVA or Mann-Whitney t-test and mean with standard deviation. * = p ≤ 0.0332; ** = p ≤ 0.002; *** = p ≤ 0.0002; **** = p ≤ 0.0001.

WU2, D39, and TIGR4 differ in their ability to form biofilms, with WU2 producing sparse biofilms with very little biomass and both D39 and TIGR4 capable of producing a robust biofilm after 24 hours of growth in a polystyrene well (Fig 4A). Similar strain-specific differences were observed in vivo with scanning-electron microscopy of nasal septum from 10-day colonized mice showing differences in the frequency and size of discernible bacterial aggregate (S5 Fig); with TIGR4 forming the largest aggregates, then D39, and last WU2. Following measurement of total IgG levels and titration for sera reactivity (S6A and S6B Fig), when we tested RAMPC_3_ mouse sera collected after the third colonization event for IgG reactivity against planktonic WCL using ELISA, we observed that reactivity was generally low across all strains (Figs 4B and S7A and S7B). In contrast, when tested against biofilm WCL, strain-specific IgG reactivity was observed with significantly greater responses for D39 and TIGR4 biofilm WCL compared to WU2 in both RAMPC_3_ cohorts (Figs 4C, S7A and S7B). Consistent with this, IgG reactivity of mouse sera to biofilm WCL positively correlated with the strain’s ability to form robust biofilms (Fig 4D). Similar patterns were observed for IgA (S7C Fig).

Given these results, we hypothesized that the strength of the host’s humoral response to asymptomatic colonization with *Spn* was dependent on the colonizing strain’s ability to form a biofilm. To test this, we colonized mice with a panel of two isogenic TIGR4 mutants previously reported, and herein re-validated, to be attenuated in their ability to form in vitro biofilms (Fig 4E) and in vivo aggregates on the surface of mucosal epithelial cells in the nasopharynx of mice during colonization (S8A and S8B Fig) [50,51]. This included *Spn* deficient in Choline-binding protein A (TIGR4∆*cbpA*), a bacterial adhesin, and Streptococcal pyruvate oxidase (TIGR4∆*spxB*), an enzyme involved in pyruvate metabolism and mixed fermentation, which is critical for conversion to the biofilm phenotype [52,53]. While the number of recoverable CFU in nasal lavage samples were not distinct between cohorts (S8C Fig), IgG reactivity of sera from these mice to biofilm WCL was reduced only for TIGR4∆*cbpA* versus wildtype (Figs 4F and S8D). As before, reactivity of these sera to planktonic TIGR4 WCL lysate was generally lower and not significantly different between colonized cohorts. SpxB modulates numerous aspects of pneumococcal physiology and host pathogen interactions due to alterations in central metabolism, capsule precursor production, hydrogen peroxide production, and autolysis. Its deletion has also been shown to alter protein production [54]. These multiple alterations potentially explain the sustained reactivity to WCL from TIGR4∆*spxB* despite its inability to form biofilms. Thus, IgG- and, to a lesser extent, IgA-based reactivity to *Spn* following asymptomatic colonization in mice generally correlate with biofilm-forming capacity, but are not solely determined by it, since additional strain-specific traits also influence host reactivity.

### The human adult humoral immune response favors recognition of the biofilm phenotype and is not capsule-dependent

**Fig 5:**
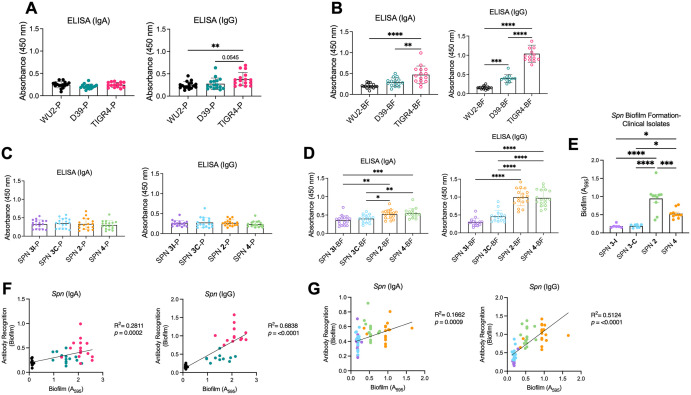
Serum antibodies from asymptomatic colonized human adults recognize *Spn* antigens depending on strain. Equal amounts of whole bacterial cell lysates (WCL) grown planktonically (P) or in a biofilm (BF) from **(A, B)** three *Spn* lab strains WU2 (serotype 3), D39 (serotype 2), and TIGR4 (serotype 4) and **(C, D)** corresponding serotype clinical isolates were run on ELISAs and individually probed with serum (1:1000) from asymptomatically colonized adults (aged 40-82) and secondary antibody α-human IgA and IgG (1:10000). Each dot is one human sample. N = 17 over one experiment. **(E)** *Spn* clinical isolates’ corresponding to the lab strains WU2, D39, and TIGR4 ability to form biofilms as measured by crystal violet assay (see methods). N = 8-10. **(F)** Linear regression correlation between ability of lab *Spn* strains and their **(G)** corresponding clinical isolates (strain-assigned colors corresponding to panels A and C, respectively) to form biofilms and antibody recognition to biofilm antigens. Mann-Whitney t-test, One-way ANOVA, and mean with standard deviation. * = p ≤ 0.0332; ** = p ≤ 0.002; *** = p ≤ 0.0002; **** = p ≤ 0.0001.

Given the translational relevance of our findings, we sought to assess their applicability in humans by examining IgA and IgG reactivity using serum from 17 adults naturally and asymptomatically colonized with *Spn* at the time of collection [55] (S3 Table). Consistent with our observations in RAMPC_3_ mice, IgA and IgG reactivity to planktonic WCL was generally low, although IgG responses to TIGR4 planktonic WCL were greater than those observed for WU2 or D39 (Fig 5A). A similar pattern was observed for IgM (S9A Fig). In contrast, IgA and IgG reactivity against biofilm WCL from TIGR4, and to lesser extent D39, was markedly higher and greater than that observed for WU2, confirming preferential reactivity in antibody recognition to proteins made during robust biofilm growth dependent on *Spn* strain (Fig 5B). This pattern was conserved when testing sera against WCL from *Spn* clinical isolates: IgA and IgG responses to planktonic WCL remained low across strains (Fig 5C), whereas IgG reactivity to biofilm WCL varied considerably, with distinct differences in recognition between strains being correlated to their ability to form biofilms (Fig 5D–5G). For these experiments, sera were also probed against purified recombinant pneumolysin (rPly) and recombinant PspA (rPspA) as positive ELISA controls, both known to be strongly immunogenic [56–59]. Antibodies against these proteins detected an IgG response that favored rPspA over rPly (S9B Fig).

Two important considerations are that our ELISA-based results likely include reactivity to capsule present in WCL, and our human samples are confounded by vaccination and prior exposures to multiple and unknown-to-us *Spn* serotypes [55]. Therefore, it was important to determine the extent to which the observed responses were due to antibody against the capsular polysaccharide. To address this, we repeated the ELISA experiments using WCL from unencapsulated, i.e., rough (R), mutant derivatives of D39, WU2, and TIGR4. Importantly, IgA and IgG reactivity to planktonic WCL remained generally equivalent across strains, and we observed no difference in reactivity between encapsulated and unencapsulated WCL (S10A and S10B Fig). The response to biofilm WCL was again stronger overall and strain dependent. This observation aligns with, for WU2 and D39, the rough derivatives producing significantly more biofilm than their encapsulated counterparts (S10C Fig), but this effect only influenced IgA reactivity to D39 and IgM to WU2 perhaps due to antibody saturation to biofilm specific proteins within the controlled conditions of our experiment. To further test for a confounding capsule effect, we used our human sera to test IgG reactivity to planktonically and biofilm-grown TIGR4 isogenic capsule switch mutant strains (TIGR4 now carrying serotypes 6D and 19A instead of serotype 4). We observed no differences in biofilm or planktonic IgG levels for the mutants compared to wildtype TIGR4 (S11 Fig). Overall, these results indicate that the preferential adult human humoral immune response to *Spn* biofilm WCL is independent of capsular polysaccharide.

### Repeated asymptomatic colonization with *Spn* confers partial protection against pneumococcal pneumonia

**Fig 6:**
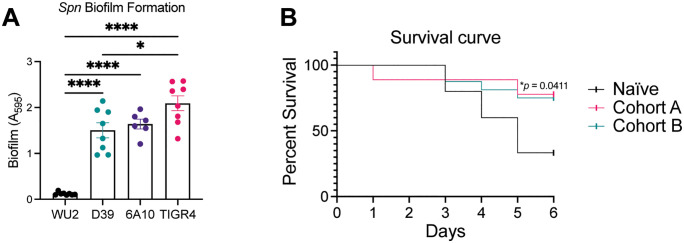
Repeated asymptomatic colonization with *Spn* partially protects against pneumococcal pneumonia. **(A)** Biofilm formation for D39, WU2, TIGR4, and 6A-10 strains as determined by crystal violet assay. N = 6-8 over one experiment. A cohort of age-matched female and male naïve mice and the RAMPC_3_ mice from both Cohorts A and B were intratracheally challenged with 10^4^ CFU of *Spn* strain 6A-10 (serotype 6A) (see methods). **(B)** Survival over time and was recorded. N = 12-15 over two experiments. Kaplan-Meier survival curve with log-rank test. One-way ANOVA and mean with standard deviation or standard error of the mean (SEM). * = p ≤ 0.0332; **** = p ≤ 0.0001.

Lastly, we sought to ascertain the extent to which triple-colonized mice were protected against pneumococcal pneumonia. To do this, RAMPC_3_ mice were infected intratracheally by forced aspiration with a fourth, unrelated *Spn* strain, 6A-10 (serotype 6A) [46], which also forms a robust biofilm (Fig 6A). Mice in both Cohorts A and B exhibited reduced mortality compared to naïve controls, confirming that asymptomatic colonization elicited a measure of protective immunity (Fig 6B). No differences in IgA or IgG as measured by ELISA for 6A-10 WCL were observed between the cohorts (S12A and S12B Fig). Along such lines, we also challenged mice previously colonized with WT TIGR4, or the biofilm-deficient mutants TIGR4Δ*cbpA* and TIGR4Δ*spxB*, with 6A-10 intratracheally and monitored for development of bacteremia. In this instance, mice formerly colonized with WT pneumococci were protected against the development of bacteremia after 24 hours of infection versus mice colonized with the biofilm deficient mutants (S13A and S13B Fig). We conclude the biofilm phenotype is an important aspect of the systemic protection that develops as result of repeated asymptomatic colonization.

### Serum IgA reactivity is comparable to mucosal tissue-derived antibody responses

Throughout our studies we had assessed IgA and IgG reactivity to pneumococcal antigens using serum. IgG is consistently and accurately measured using this method, providing a comprehensive view of the systemic humoral immune response during sequential colonization. This contrasts with IgA, the predominant mucosal-associated immunoglobulin found in airway secretions and tissue-specific sites such as nasal-associated lymphoid tissue (NALT) in mice or Waldeyer’s ring in humans [60]. To determine whether IgA derived from mucosal tissue mirrored our serum findings, we measured NALT IgA reactivity in three murine cohorts. Each cohort was colonized once with either WU2, D39, or TIGR4 and tested for reactivity against planktonic and biofilm cell lysates using ELISA. We observed similar patterns of IgA reactivity to biofilm bacterial lysates from NALT as those seen in serum (S14A–S14C Fig).

## Discussion

Recent conjugate vaccines targeting the *Spn* polysaccharide capsule have greatly reduced childhood incidence and mortality from pneumococcal infection. However, *Spn* remains a leading cause of community-acquired pneumonia, underscoring the need for improved protective strategies [61]. Because colonization is a prerequisite for disease development, understanding the interactions *Spn* has with the host during colonization is critical for identifying or prioritizing antigens for next-generation protein-based vaccines [3]. Our study demonstrates, using a novel repeated-colonization mouse model and human sera, that systemic humoral immunity strongly recognizes proteins produced preferentially during biofilm growth. What is more, we show the first colonizing *Spn* strain imprints a stable pattern of antibody recognition that persists through subsequent colonization. In turn, this confers partial protection against pneumonia-challenge with other strains belonging to other serotypes.

Over the past 25 years, *Spn* biofilm formation has gained recognition for its role in enhancing adherence to mucosal epithelial cells during colonization, promoting survival on fomites, and facilitating bacterial interactions that increase competence and genetic exchange [30,32,34,35,62]. However, most immunological studies have focused on planktonic antigens with their more commonly known impact on disease, overlooking that *Spn* typically exists as a biofilm during carriage [63]. These differences are considerable as one study identified a 30% difference in the detectable proteome when comparing planktonic cultures versus that of 3-day old biofilms, with 200 newly synthesized proteins, and 14% of the proteome upregulated and 18% downregulated [64]. Additionally, the difference between total possible proteins being produced during bacterial growth increased 30% when *Spn* was grown as a biofilm versus planktonically. Many of these upregulated proteins are involved with carbon metabolism, virulence, and surface association and have been reported by other groups [65]. We speculate the enlarged proteome during biofilm growth is reflective of the growth conditions present, with upregulation of genes encoding these proteins needed to overcome the stressors associated with biofilm culture. This aspect of pneumococcal biology may provide an opportunity for a prioritization of antigens in context of vaccines that seek to prevent or diminish colonization, which is requisite for disease.

Our findings with sera from colonized mice confirm that asymptomatic pneumococcal colonization induces a systemic humoral response to protein antigens. This was consistent with previous reports [49,66] and aligns with controlled human infection studies in which subjects with higher antibody levels against pneumococcal proteins were better protected against rechallenge [67]. We did not identify specific proteins recognized by human sera due to unknown prior exposure history. However, antibodies in sera obtained from repeatedly colonized mice targeted surface adhesins (PhtD, PsaA, PspA, and a subtilisin-family serine protease) and cell wall hydrolases (LysM and lytic transglycosylase G). Cross-referencing these genes with in vivo RNA-seq data confirmed their expression was enhanced by *Spn* during nasopharyngeal colonization [42]. Many of these protein classes overlap with antigens recognized by IgG in other human serological studies of children and adults [20,27,68]. While proteins such as PspA and PsaA have been extensively characterized as protective antigens [13,69–74], to the best of our knowledge they have not been specifically evaluated for their ability to reduce colonization duration or bacterial burden during repeated colonization with heterologous strains. Our findings suggest that such efforts are warranted and that the RAMPC_3_ model is well-suited to them. Critically, the heterogeneity of highly antigenic pneumococcal proteins such as PspA, which can be divided into two serologically distinct “families”, likely strongly influences the efficacy of the host response to subsequent *Spn* colonization [75]. Broader recognition of different versions of these proteins most likely develops over repeated colonization events with distinct strains.

We developed the RAMPC_3_ model to create a multi-strain, multi-colonization system that better reflects human exposure while allowing control over strain order. RAMPC_3_ findings showed that nasopharyngeal bacterial burden depended on both the number of colonization events and the strain order; these factors are critical for understanding heterologous cross-protection and disease prevention [76]. Our results with RAMPC_3_-generated sera mirrored data obtained with human sera, showing strong recognition of biofilm antigens, and further validating the model. Because our laboratory strains were well characterized, we were able to link host response strength to each strain’s ability to form robust biofilms in vitro and in vivo. We also had the ability to challenge mice with a fourth strain to assess the impact of colonization on infection and confirmed that IgA responses in NALT reflected those observed in serum.

Importantly, protection against lethal intratracheal challenge after repeated asymptomatic colonization was not absolute and a meaningful number of mice still succumbed to the infection. However, mice that were colonized with biofilm-deficient pneumococci were more likely to develop bacteremia following heterologous intratracheal challenge than mice initially colonized with wildtype bacteria, suggesting that the biofilm-focused response was not marginal. Interestingly, and unlike TIGR4Δ*cbpA*, following colonization by TIGR4Δ*spxB* there was still elevated IgG recognition to WT TIGR4 biofilm WCL. Because of the multifactorial role of SpxB it is difficult to pinpoint the reason behind these results [77–80]. Although SpxB deficiency is associated with decreased capsule, this is unlikely to be an explanation as we observed no difference in sera reactivity following colonization with rough and encapsulated variants. Importantly, and despite reactivity to biofilm WCL being unchanged as result of *spxB* deletion, our data still strongly supports the notion that the host response to asymptomatic colonization, both in mice and humans, is primarily to biofilm-associated antigens in manner that is dependent on the initial colonizing *Spn* strain, and this confers a degree of protection.

It is necessary to consider the contributions from the other arm of the adaptative immune system, particularly IL-17 producing T-cells [14,18]. Several works by Mizgerd and colleagues have shown a protective role for CD4 + resident memory T cells during repeated pneumococcal pneumonia [15,81,82]. Future experiments are required to delve more thoroughly into this topic and to explore the implications of asymptomatic carriage versus disease on development of cell-mediated immunity. It is possible that key protective T-cell antigens are also biofilm-associated. Notably, the notion that biofilm antigens could be protective aligns with recent work by Jensen *et al.* who introduced the concept of biofilm-associated molecular patterns (BAMPs) as unique immunostimulatory molecules highly expressed in biofilms [83].

Another important consideration of our study is that pneumococci forming biofilms in vitro are likely quite different than pneumococci forming aggregates or biofilms in vivo. Herein, we infer heavily on differences observed between reactivity of sera to planktonic WCL and biofilm WCL, both grown in vitro and collected at different growth stages. Nonetheless, following repeated colonization reactivity of collected sera to biofilm WCL is greater than to planktonic WCL suggesting that key aspects that occur during *Spn* biofilm formation within a polystyrene well are also occurring during colonization and impacting the host. However, care must be taken when interpreting specific facets of the in vitro condition, such as upregulation of key antigens, due to its arbitrary nature, and in vivo validation remains a necessity.

In conclusion, our study provides new insights into pneumococcal carriage and disease, highlighting how biofilms influence humoral immunity and potentially other aspects of protection by using the novel triple colonization RAMPC_3_ model. Although the concept of “original antigenic sin” is well established in viral infection, its role in bacterial pathogenesis is not well appreciated with limited evidence of its occurrence or impact on outcomes regarding repeated infections. Our findings underscore that the biofilm phenotype plays a dominant role during colonization and is the primary version of the bacteria recognized by the humoral response against *Spn*. Collectively, this information has the potential to be leveraged regarding antigen selection for future vaccine formulations.

## Materials & methods

### Ethics statement

Human serum samples from adult volunteers aged 40–82 years were collected under Institutional Review Board (IRB) approved protocols (CUS 01–20Feb2020, Shaio 301–26Aug2020, IPS-Clínicos 01–20Feb2020, and Baxter 05–01Sep2021) at the Centro de Investigación Unisabana Center for Translational Science at the Universidad de La Sabana in Chia, Colombia (see S2 Table). Written formal consent was obtained. Serum samples were de-identified and did not meet the criteria for human subject research [55]. Animal experiments were performed under the Institutional Animal Use and Care Committee (IACUC) approved protocol #22157 at The University of Alabama at Birmingham. Animal care and experimental protocols adhered to Public Law 89–544 (Animal Welfare Act) and its amendments, Public Health Services guidelines, and the Guide for the Care and Use of Laboratory Animals (U.S. Department of Health & Human Services).

### Bacterial strains

Several strains of *Spn* were used in this study (see S1 Table). All bacteria were grown from frozen stock on tryptic soy agar plates with 5% sheep blood at 37°C and 5% CO_2_ overnight. Working broth cultures were grown in Todd-Hewitt broth with 0.5% yeast extract (THY) to exponential phase (OD_621_: ~ 0.3-0.5) before being serially diluted in saline for experiments or harvested for protein. For 24-hour biofilm growth, a 1:100 culture of *Spn* was grown in THY in a sterile 100mm polystyrene plate at 37°C and 5% CO_2_ overnight.

### Bacterial lysis and protein quantification

For planktonic *Spn* lysates, 1 mL aliquots of bacterial cultures were spun down at ~2,500xG for 3 minutes, then resuspended in protein lysis buffer (50mM Tris, 150mM NaCl, 1% TritonX-100) with 10% sodium dodecyl sulfate (SDS) and deoxycholate added in phosphate buffer saline (PBS). Lysates were incubated at 37°C and 5% CO_2_ until the liquid was clear. Biofilm *Spn* were washed twice with PBS after THY was removed, collected by washing with 1 mL of PBS, spun down and resuspended in protein lysis buffer, and then lysed as stated above. Protein concentration was quantified using the Pierce BCA Protein Assay Kit per the manufacturer’s recommendation (Thermo Scientific, #23225).

### Recombinant protein purification

Purification of recombinant proteins pneumolysin (rPly) and PspA (rPspA) was performed by using cobalt-affinity resin and the *E. coli* strains BL21(DE3) and NEBExpress Iq carrying the respective plasmids [84,85]. The strains were grown at 37°C in Luria-Bertani (LB) broth with required antibiotics, and expression was induced at OD_621_ = 0.4 by the addition of 1 mM isopropyl β-D-1-thiogalactopyranoside (IPTG). After 4 hours of induction, cells were harvested and lysed in BugBuster Master Mix (MilliporeSigma, #71456–4) with 1 mM phenylmethylsulfonyl fluoride (PMSF) for 30 minutes at room temperature with gentle shaking. The cell lysates were spun at ~3,000xG for 20 minutes, and the supernatant was loaded on beads pre-equilibrated with default buffer (5 mM Imidazole, 50 mM Tris-HCl pH 7.5, 150 mM NaCl). Beads were washed 10 times with the default buffer, and protein was eluted in the default buffer containing 100 mM imidazole. Collected protein fractions were confirmed on an SDS-PAGE gel and concentrated/buffer exchanged to PBS using a centrifugal concentrator tube. Protein concentration was measured by using the Pierce BCA Protein Assay Kit per the manufacturer’s recommendation. Equal loading of lysates was confirmed by Coomassie staining of SDS-PAGE gel and Ponceau staining of transferred proteins on nitrocellulose membrane.

### Immunoblots

Whole-cell bacterial lysates (WCL) (10 μg/150 μL) and recombinant protein (rPly: 0.1 μg/150 μL, rPspA: 1 μg/150 μL) were mixed with NuPAGE LDS Sample Buffer (4X) (ThermoFisher, NP0007) and β-mercaptoethanol before denaturing for 10 minutes at 95°C for 10 minutes. Samples were loaded onto an SDS-PAGE gel (4–15%; BioRad) before transferring onto a nitrocellulose membrane. After blocking with 5% bovine albumin serum (BSA), the membrane was incubated overnight at room temperature with the primary antibody, followed by washing with 1% tris-buffered saline-tween (TBS-Tween20) and incubation with the secondary antibody. Visualization of the blots was done using the Pierce ECL Western Blotting Substrate (ThermoFisher, PI32209) and ChemiDoc XRS+ System (BioRad). Primary antibody: human Colombia patient cohort (1:1000) or mouse serum (1:1000). Mouse NALT analysis (1:100). Secondary antibodies: HRP-conjugated goat α-human IgA (Invitrogen, #PA1–74395), HRP-conjugated goat α-human IgG (Jackson Immuno Research, #109-035-088), HRP-conjugated goat α-mouse IgA (Abcam, #ab97235), HRP-conjugate goat α-mouse IgG (Invitrogen, #31430) (all at 1:10,000).

### Detection of immunoglobulin protein band patterns via immunoblot

*Spn* whole-cell lysates were probed with serum from colonized mice and reactive protein bands were quantified for each immunoblot following the first colonization event in Cohorts A and B. Bands detected after the second and third colonization events that had been previously identified were excluded to quantify only newly detected reactive bands. These values were plotted as the “number of newly detected proteins” for IgA or IgG based reactivity relative to the first colonization event.

### ELISA (enzyme-linked immunosorbent assay)

For detection of human and mouse IgA and IgG responses to various *Spn* lysates, a 96 well plate (ThermoFisher Immulon 4HBX, #3855) was coated with 2 μg/mL of rPly, rPspA, and the planktonic and biofilm-grown lysates of WU2, D39, TIGR4, and 6A-10 in PBS, covered, and placed at 4°C overnight. The next day, the plate was washed three times with PBS-Tween before blocking with 5% bovine albumin serum (BSA) in PBS for 1 hour at room temperature. The plate was washed twice more as above before the addition of desired primary serum diluted in PBS at (1:1000) and incubated for 3 hours at room temperature. After incubation with the primary serum, the plate was washed three times before incubation with the desired secondary antibody diluted in PBS at (1:10,000) and incubated for 1 hour at room temperature. The plate was washed five times before the addition of tetramethylbenzidine (TMB) substrate reagent (BD OptEIA TMB Substrate Reagent Kit, #555214) to each well-prepared according to the manufacturer’s recommendation and incubated in the dark for 10 minutes at room temperature. 2N sulfuric acid was added to each well to stop the reaction, and the absorbance was read on a BioTek Cytation 5 Cell plate reader at 450 nm. Primary antibody: human (Colombia patient cohort) or mouse sera (1:1000). Mouse NALT analysis (1:10). Secondary antibodies: HRP-conjugated goat α-human IgA (Invitrogen, #PA1–74395), HRP-conjugated goat α-human IgG (Jackson Immuno Research, #109-035-088), HRP-conjugated goat α-mouse IgA (Abcam, #ab97235), HRP-conjugated goat α-mouse IgG (Invitrogen, #31430) (1:10,000). To quantify total IgG in mouse serum samples, plates were coated with normal mouse IgG (SouthernBiotech #0107–14) serially diluted in PBS to establish a standard curve, or with serum diluted 1:20000 in PBS. Following incubation at 4°C overnight, the ELISA was performed as above but without the addition of a primary antibody.

### Crystal violet assay for biofilm quantification

*Spn* lab and clinical strains of interest were grown as biofilms, as described above. After 24 hours, the media and planktonic bacteria were carefully aspirated, and the remaining biofilm layer was washed three times with PBS. The washed biofilms were allowed to dry completely before incubation with 0.1% crystal violet (Sigma-Aldrich, #C3886) stain in distilled water for 20 minutes at room temperature. The stain was removed, and the biofilms were washed as described. After completely drying again, the stained biofilms were resolubilized (95% ethanol and 5% acetic acid), and dilutions were added to a 96-well plate. The absorbance was read on a BioTek Cytation 5 Cell plate reader at 595 nm.

**Repeated Asymptomatic Murine Pneumococcal Colonization (RAMPC**_**3**_**) Model** C57BL/6J female and male mice aged ~9 weeks (The Jackson Laboratory) in Cohort A were inoculated intranasally with *Spn* strain WU2 (10^4^ CFU). At various timepoints (1, 3, 7, and 14 days), 10 μL of PBS was used to wash the nasal cavity of each mouse while under isoflurane sedation. ~ 2 μL of recovered liquid was then diluted ten-fold, mixed thoroughly with a pipette, and plated onto blood agar plates for colony forming unit (CFU) enumeration. After ~1 month, mice were inoculated with D39, and nasal washes were repeated as described. Another month later, mice were inoculated with TIGR4. Serum from mice was collected via retro-orbital eye bleeds on days -7, 21, 49, and 79. Mice in Cohort B were colonized in the same manner, except the order of strains was TIGR4, D39, and WU2. Mice for NALT analysis were colonized once using the same method with D39, WU2, and TIGR4. NALT was harvested after ~2 weeks, as described previously [86] and homogenized in 1 mL of PBS.

### Biofilm-deficient mutant colonization and ex vivo biofilm quantification

C57BL/6J female and male mice aged 8–10 weeks (The Jackson Laboratory) were inoculated intranasally as above with *Spn* strains TIGR4, TIGR4∆*cbpA*, or TIGR4∆*spxB* (10^4^ CFU), with ten mice per group. At 7 and 10 days, 10 µL of PBS was used to wash the nasal cavity of each mouse while under isoflurane sedation. ~ 2 µL of recovered liquid was diluted ten-fold in PBS, and then 10 µL of the suspension was combined 1:1 with 1% crystal violet in H_2_O (Sigma-Aldrich, #C3886). 10 µL of this mixture was transferred to a glass slide, a coverslip was applied, and bacterial aggregates were counted at 1000x magnification using a Leica CME microscope [31,37]. 50–100 aggregates were counted per sample and classified as 1, 2–9, or ≥10 diplococci. Number of aggregates for each of these three categories were expressed as a percentage of the total number of aggregates. On day 21 post-colonization, serum from mice was collected via retro-orbital eye bleeds.

### In vivo infections

C57BL/6J female and male mice aged ~9 weeks (The Jackson Laboratory) or age-matched mice for the repeated colonization model were sedated using 2.5% vaporized isoflurane in oxygen. For intratracheal challenge (pneumonia model), the inoculum was prepared in PBS, and 100 μL containing 10^3-4^ CFU was instilled into the lower airways by forced aspiration [87]. All animals were observed for recovery following infection and monitored daily afterward. Bacteremia was determined using blood obtained by tail bleed (~2 μL of blood per mouse) diluted in PBS and enumerated via CFUs on blood agar plates. Serum was collected from surviving mice by retro-orbital eye bleeds.

### Pneumococcal protein array

A *Spn* protein array containing 254 proteins was constructed. Proteins were selected based on having a high level of conservation in a panel of >600 *Spn* strains and included the majority of the conserved proteins that were significantly recognized by IgG present in human sera obtained from healthy adults [68]. The array was constructed using genes amplified from bacterial genomic DNA (*Spn* strain TIGR4) and cloned into a T7 expression vector. Proteins were expressed by incubating the plasmids for 16 hours in *E. coli*-based *in vitro* transcription/translation (IVTT) reactions (RTS E. coli HY 100 kit, biotechrabbit, #BR1400106). Proteins were tested for expression by immunoblot using antibodies against N-terminal poly-histidine (His) after transfer onto nitrocellulose-coated glass AVID slides (Grace Bio-Labs, #305384), using an Omni Grid 100 microarray printer (Genomic Solutions). Arrays were probed with mouse serum samples diluted (1:100) in protein array blocking buffer (GVS, #10485356) and supplemented with *E. coli* lysate. Images were acquired using a Innoscan 710G scanner (Innopsys) and analyzed using Mapix 8.5 software. “No DNA” controls consisting of *E. coli* IVTT reactions without the addition of DNA were averaged and used to subtract background *E. coli* reactivity. All results presented are expressed as Mean Fluorescence Intensity (MFI).

### In vivo gene expression of *Spn* biofilm-specific antigens in mouse tissues

See D’Mello *et al.* 2020 for full detailed methods [42]. Mice were colonized with 10^5^ CFU or intraperitoneally infected with 10^4^ CFU of *Spn* strains D39 (serotype 2), TIGR4 (serotype 4), and 6A-10 (serotype 6A). RNA was isolated from the nasopharynx or the blood post-infection and prepared for sequencing. Comparison of gene expression of the six antigens identified from the *Spn* protein array (PhtD, LysM, PsaA, LtG, SP, and PspA) were made in the nasopharynx versus the blood. All genes are differentially expressed (DE) as determined by log2 fold changes and false discovery rates.

### Scanning electron microscopy (SEM)

C57BL/6J female mice aged 8–10 weeks (The Jackson Laboratory) were inoculated intranasally with either WU2, D39, TIGR4, TIGR4∆*cbpA*, or TIGR4∆*spxB* as described above. On day 10 after inoculation, mice were euthanized, exsanguinated, and perfused with 3 mL of PBS, and nasal septa were excised as previously described [88], with the exception that the cuts to expose the septum were made after fixation. Samples were fixed overnight in Karnovsky’s fixative: 5% paraformaldehyde (Electron Microscopy Sciences #15710), 4% glutaraldehyde (Electron Microscopy Sciences #16300), and 0.1 M sodium cacodylate (Electron Microscopy Sciences #11652). Samples were processed for SEM using a HMDS dehydration strategy as previously described [89]. For all microwave steps, a microwave processor (PELCO #36700) set at 100 W was used. Samples were platinum/palladium sputter coated prior to imaging. Images were captured on a JEOL JSM-IT810 SEM.

### Statistical analysis

All statistical analyses were done using GraphPad Prism (version 10). For comparisons of two groups, the nonparametric two-tailed Mann-Whitney log-rank *t*-test was used, and the median with a 95% confidence interval (CI) or the standard deviation (SD) is shown. For all multiple comparisons, a nonparametric one-way or two-way analysis of variance (ANOVA) with Tukey’s pairwise test was used, and the SD is shown.

## Supporting information

S1 Fig *Spn* D39 burden in a murine asymptomatic colonization model. (PDF)

S2 Fig Planktonic and biofilm WCL profiles from three different *Spn* strains and naïve sera from mice prior to repeated *Spn* colonization. (PDF)

S3 Fig The first colonization event imprints a mucosal and systemic antibody response to the proteins that persist following repeated colonization. (PDF)

S4 Fig In vivo gene expression of *Spn* biofilm-specific antigens in mouse tissues and *Spn* protein array. (PDF)

S5 Fig SEM *Spn* biofilm formation on murine nasal septa differs depending on strain and biofilm capabilities. (PDF)

S6 Fig Total serum IgG and dilution curves from RAMPC_3_ cohorts. (PDF)

S7 Fig Repeated murine *Spn* colonization elicits a strain-dependent humoral response associated with biofilm formation. (PDF)

S8 Fig In vivo biofilm aggregate formation is dependent on *Spn* strain. (PDF)

S9 Fig Serum antibodies from asymptomatic colonized human adults recognize *Spn* antigens depending on strain. (PDF)

S10 Fig *Spn* antigen immunoglobulin specificity in asymptomatic adult carriers is not capsule-dependent. (PDF)

S11 Fig Serum antibodies from asymptomatic colonized human adults recognize *Spn* antigens regardless of capsule type in the same genetic background. (PDF)

S12 Fig Repeated asymptomatic colonization with *Spn* partially protects against pneumococcal pneumonia. (PDF)

S13 Fig *Spn* deficient in biofilm formation are less protected against pneumococcal pneumonia following colonization. (PDF)

S14 Fig Serum IgA reactivity is comparable to mucosal tissue-derived antibody responses. (PDF)

S1 Raw Image Supporting raw images of gels and blots. (PDF)

S1 Table *Streptococcus pneumoniae* strains used throughout the study. See methods and references for details.
(DOCX)

S2 Table Pneumococcal protein array raw data (as mean fluorescence intensity). Antigens are listed in column A in SP gene annotation. Columns B-O (light pink) are from Cohort A mice and Columns P-AC (light blue) are from Cohort B mice. 1^st^ versus 3^rd^ delineates serum collected after the first or third colonization. Color coding is red > 50000, black>25000 and white = 0 MFI. < 5000 MFI is not significant.
(XLSX)

S3 Table Deidentified metrics from human individuals naturally colonized with *Streptococcus pneumoniae.* See reference Olivella-Gomez *et al.* 2025.
(DOCX)
