# Supplementary material for: Biofilm formation during pneumococcal carriage imprints naturally acquired humoral immunity
Source: PLoS Pathog. 2026 Jul 28;22(7):e1013826. doi: 10.1371/journal.ppat.1013826 (PMC13426961; doi:10.1371/journal.ppat.1013826)
Supplement: S1 Fig — (PDF) [file ppat.1013826.s001.pdf]

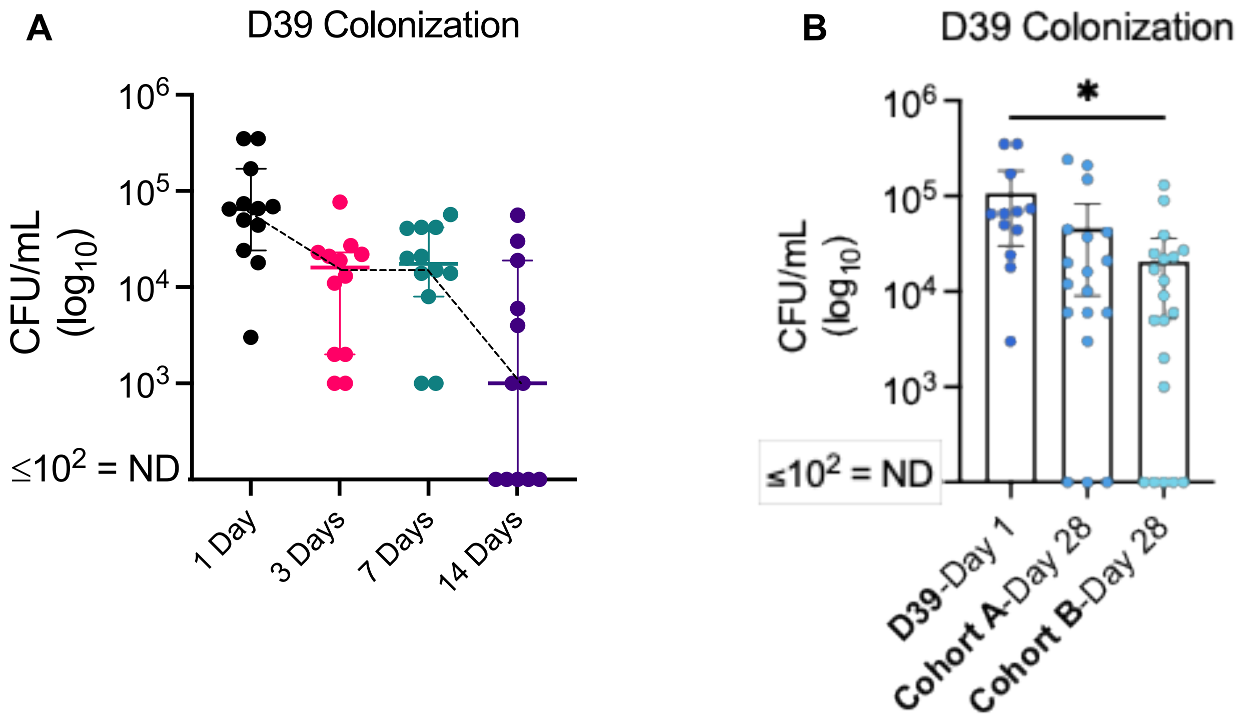

S1 Fig. *Spn* D39 burden in a murine asymptomatic colonization model. (A) 9-week-old C57BL/6J male and female mice were intranasally inoculated with 10^4 CFU of D39 (serotype 2). Bacterial burden was determined over a 2-week period post-inoculation by colony forming units (CFUs) obtained from nasal washes with saline. N=12 per group over one experiment. **(B)** CFUs from D39 colonized mice after single infection of Cohort A or Cohort B of the $RAMPC_3$ model one day after colonization. Each dot is one mouse sample. Not detected (ND) ≤ 10^2 CFU/mL. One-way ANOVA and median with 95% confidence interval (CI). *=p≤0.0332.
