# Supplementary material for: Biofilm formation during pneumococcal carriage imprints naturally acquired humoral immunity
Source: PLoS Pathog. 2026 Jul 28;22(7):e1013826. doi: 10.1371/journal.ppat.1013826 (PMC13426961; doi:10.1371/journal.ppat.1013826)
Supplement: S2 Fig — (PDF) [file ppat.1013826.s002.pdf]

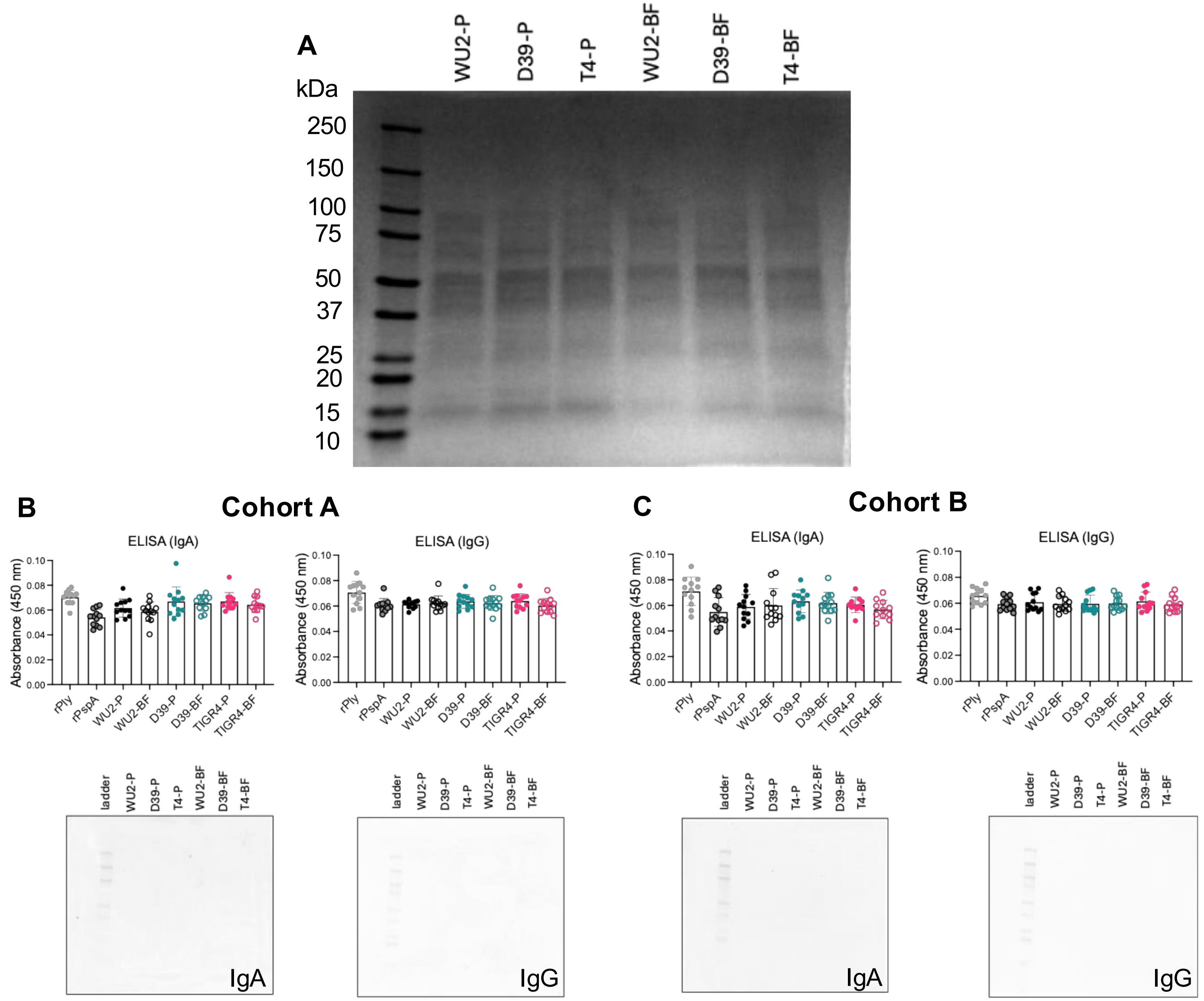

S2 Fig. Planktonic and biofilm WCL profiles from three different *Spn* strains and naïve sera from mice prior to repeated *Spn* colonization. (A) Recombinant (r) protein and equal amounts of whole bacterial cell lysates (WCL) grown planktonically (P) or in a biofilm (BF) from three *Spn* lab strains WU2 (serotype 3), D39 (serotype 2), and TIGR4 (serotype 4) were run on an SDS-PAGE gel. The gel was stained with Coomassie Blue (see methods). Equal amounts of whole bacterial cell lysates grown planktonically (P) or in a biofilm (BF) from three *Spn* strains WU2 (serotype 3), D39 (serotype 2), and TIGR4 (serotype 4) were analyzed by immunoblot. Membranes were probed individually with mouse sera (1:1000) from **(B)** Cohort A and **(C)** Cohort B RAMPC$_3$ mice prior to colonization. Representative blots shown. Recombinant (r) protein and equal amounts of whole bacterial cell lysates grown planktonically (P) or in a biofilm (BF) from three *Spn* strains WU2 (serotype 3), D39 (serotype 2), and TIGR4 (serotype 4) were run on ELISAs and probed using serum (1:1000) from RAMPC$_3$ mice in both Cohort A and Cohort B prior to colonization. Secondary antibody α-mouse IgA and IgG (1:10000). Each dot is one mouse sample. N=12 over one experiment. Standard deviation is shown.
