# Supplementary material for: Biofilm formation during pneumococcal carriage imprints naturally acquired humoral immunity
Source: PLoS Pathog. 2026 Jul 28;22(7):e1013826. doi: 10.1371/journal.ppat.1013826 (PMC13426961; doi:10.1371/journal.ppat.1013826)
Supplement: S3 Fig — (PDF) [file ppat.1013826.s003.pdf]

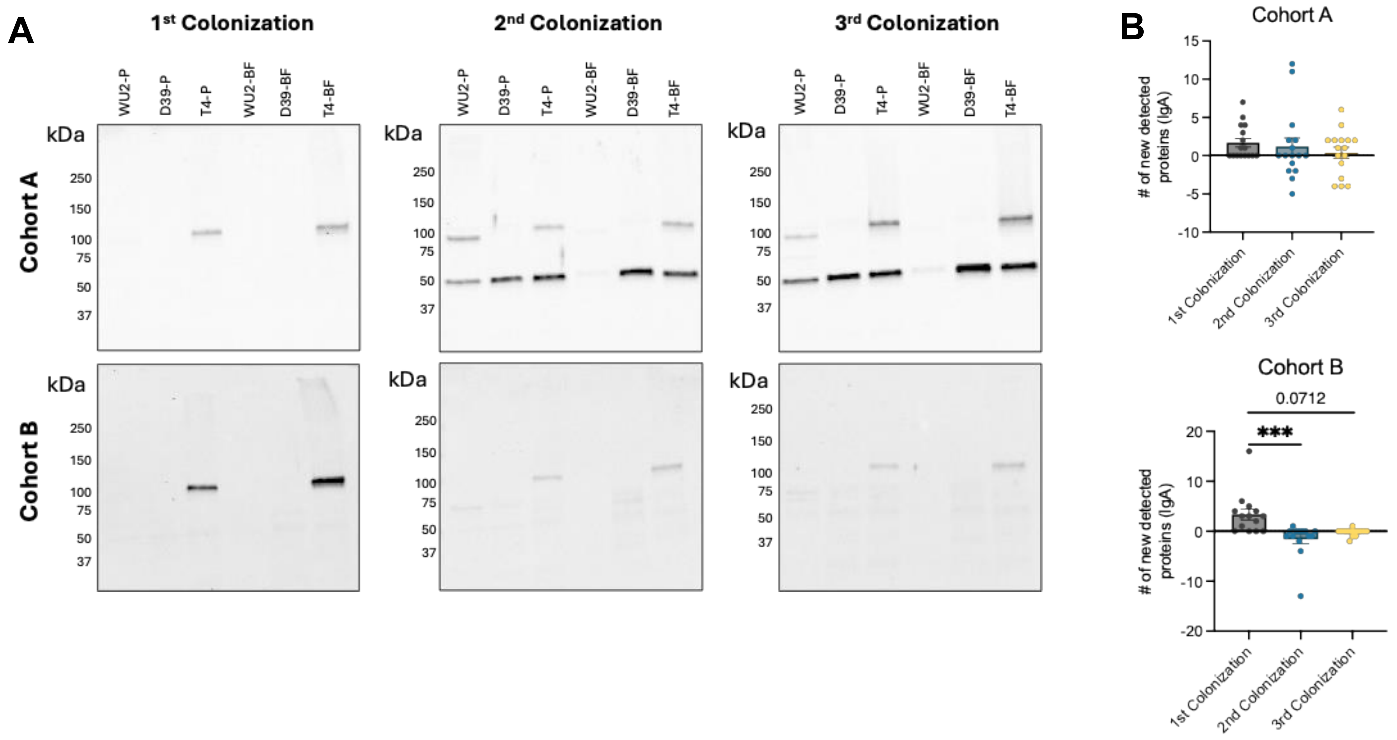

S3 Fig. The first colonization event imprints a mucosal and systemic antibody response to the proteins that persist following repeated colonization. (A) Equal amounts of whole bacterial cell lysates grown planktonically (P) or in a biofilm (BF) from three *Spn* strains WU2 (serotype 3), D39 (serotype 2), and TIGR4 (serotype 4) were analyzed by immunoblot. Membranes were probed individually with mouse sera (1:1000) from Cohort A and Cohort B RAMPC$_3$ mice after the first, second, and third colonization events and secondary α-mouse IgA (1:10000). Representative blots shown. **(B)** The number of new protein antigens detected by IgA on immunoblots from Cohort A and Cohort B RAMPC$_3$ mice after the first, second, and third colonization events (see methods). N=15-16 over two separate experiments. One-way ANOVA and mean with standard deviation. ***=p $\leq$0.0002.
