# Supplementary material for: Biofilm formation during pneumococcal carriage imprints naturally acquired humoral immunity
Source: PLoS Pathog. 2026 Jul 28;22(7):e1013826. doi: 10.1371/journal.ppat.1013826 (PMC13426961; doi:10.1371/journal.ppat.1013826)
Supplement: S4 Fig — (PDF) [file ppat.1013826.s004.pdf]

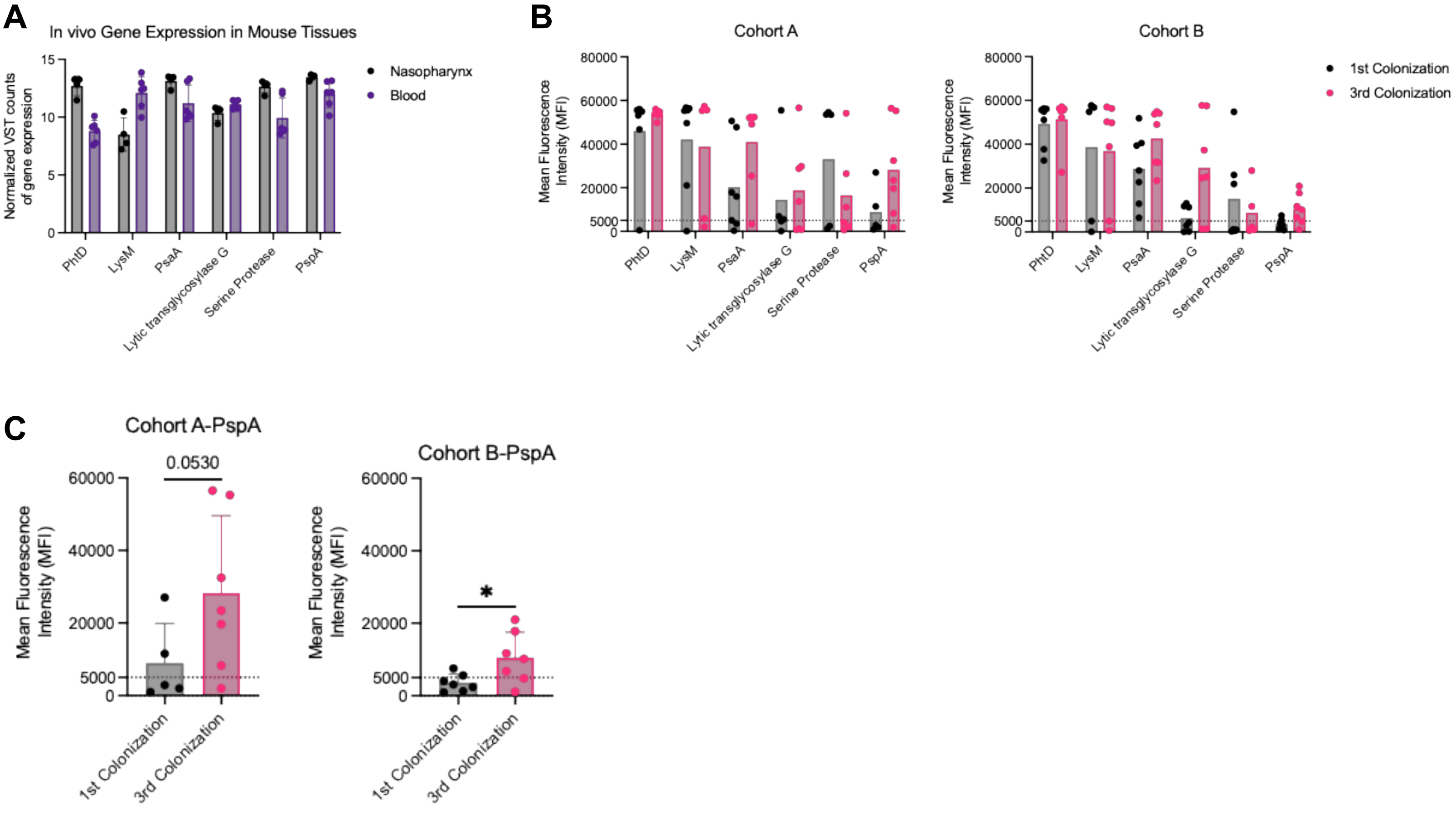

S4 Fig. In vivo gene expression of *Spn* biofilm-specific antigens in mouse tissues and *Spn* protein array. Mice were either colonized at 10^4 CFU or intraperitoneally infected at 10^4 CFU with *Spn* strains D39 (serotype 2), TIGR4 (serotype 4), and 6A-10 (serotype 6A). RNA was isolated from the nasopharynx or the blood post-infection and prepared for sequencing (D'Mello *et al.* 2020). **(A)** Comparison of gene expression of the six antigens identified from the *Spn* protein array (PhtD, LysM, PsaA, LtG, SP, and PspA) in the nasopharynx versus the blood. All genes are differentially expressed (DE) as determined by log2 fold changes and false discovery rates. N=4-6 over one experiment. Each dot is one mouse sample. **(B)** A pneumococcal protein array was constructed with 254 highly antigenic proteins. Proteins were selected from a panel of *Spn* strains and were conserved for recognition by IgG from healthy human adults (Croucher *et al.* 2017). Sera from $RAMPC_3$ colonized mice after the first and third colonization events were used to probe the protein array for each cohort (1:100) (see methods). N=5-7 per Cohort over two separate experiments. Each dot is one mouse sample. Limit of detection was MFI=5000. **(C)** MFI of PspA for Cohort A and Cohort B after the first and third colonization. Mann-Whitney t-test and mean with standard deviation. *=p≤0.0332.
