# Supplementary material for: Biofilm formation during pneumococcal carriage imprints naturally acquired humoral immunity
Source: PLoS Pathog. 2026 Jul 28;22(7):e1013826. doi: 10.1371/journal.ppat.1013826 (PMC13426961; doi:10.1371/journal.ppat.1013826)
Supplement: S5 Fig — (PDF) [file ppat.1013826.s005.pdf]

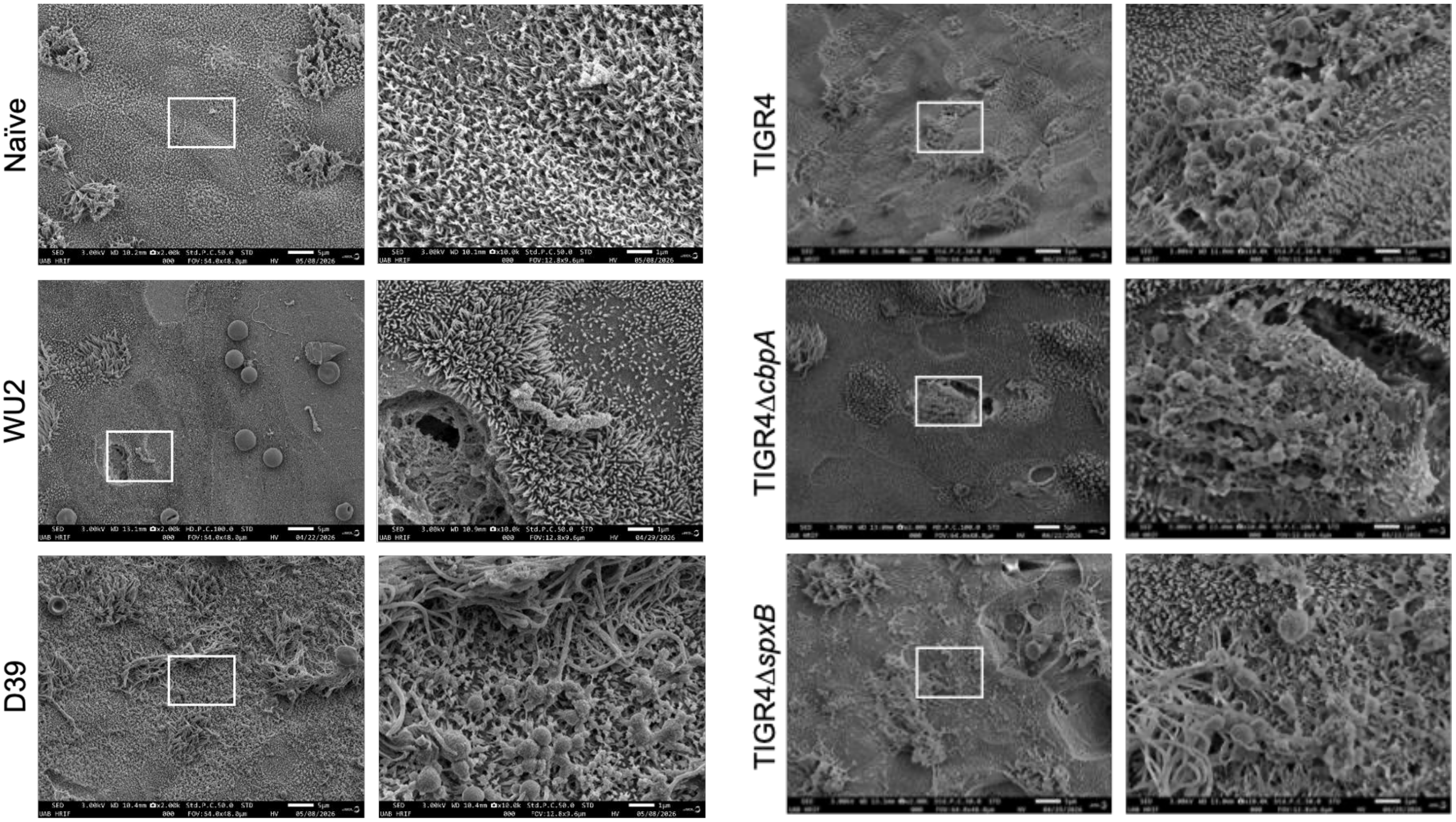

S5 Fig. SEM *Spn* biofilm formation on murine nasal septa differs depending on strain and biofilm capabilities. Mice were colonized (10^4 CFU) with *Spn* strains WU2 (serotype 3), D39 (serotype 2), TIGR4 (serotype 4), and biofilm-deficient mutants in the TIGR4 background (TIGR4Δ*cbpA* and TIGR4Δ*spxB*). 10 days post-colonization, nasal septa were harvested, fixed, and prepared for scanning electron microscopy (SEM) (see methods). Images are shown at 2000x magnification (left image) with the white box zoomed in at 10000x (right image) magnification. N=1 per sample over one experiment.
