# Supplementary material for: Biofilm formation during pneumococcal carriage imprints naturally acquired humoral immunity
Source: PLoS Pathog. 2026 Jul 28;22(7):e1013826. doi: 10.1371/journal.ppat.1013826 (PMC13426961; doi:10.1371/journal.ppat.1013826)
Supplement: S6 Fig — (PDF) [file ppat.1013826.s006.pdf]

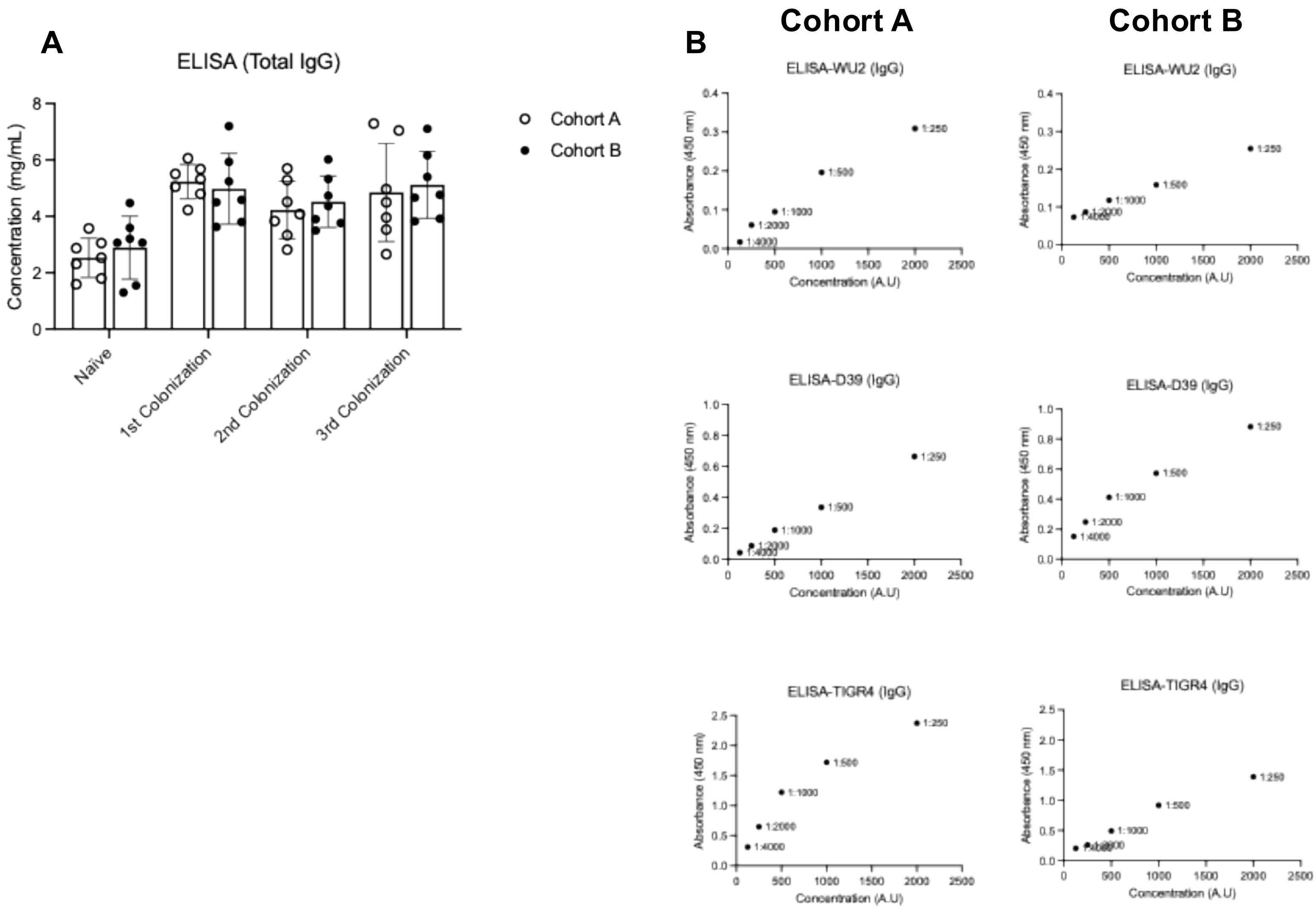

S6 Fig. Total serum IgG and dilution curves from RAMPC$_3$ cohorts. (A) An ELISA plate was coated with normal mouse IgG serially diluted in PBS to establish a standard curve and with RAMPC$_3$ serum from Cohort A and Cohort B (1:20000). The primary antibody was PBS and the secondary antibody was α-mouse IgG (1:10000). **(B)** Equal amounts of whole bacterial cell lysates grown in a biofilm (BF) from three *Spn* strains WU2 (serotype 3), D39 (serotype 2), and TIGR4 (serotype 4) were run on ELISAs and probed using serum at various dilutions (1:250-1:4000) from RAMPC$_3$ mice in both Cohort A and Cohort B following colonization. Secondary antibody α-mouse IgG (1:10000). Each dot is one mouse sample. N=7 over one experiment. Standard deviation is shown.
