# Supplementary material for: Biofilm formation during pneumococcal carriage imprints naturally acquired humoral immunity
Source: PLoS Pathog. 2026 Jul 28;22(7):e1013826. doi: 10.1371/journal.ppat.1013826 (PMC13426961; doi:10.1371/journal.ppat.1013826)
Supplement: S7 Fig — (PDF) [file ppat.1013826.s007.pdf]

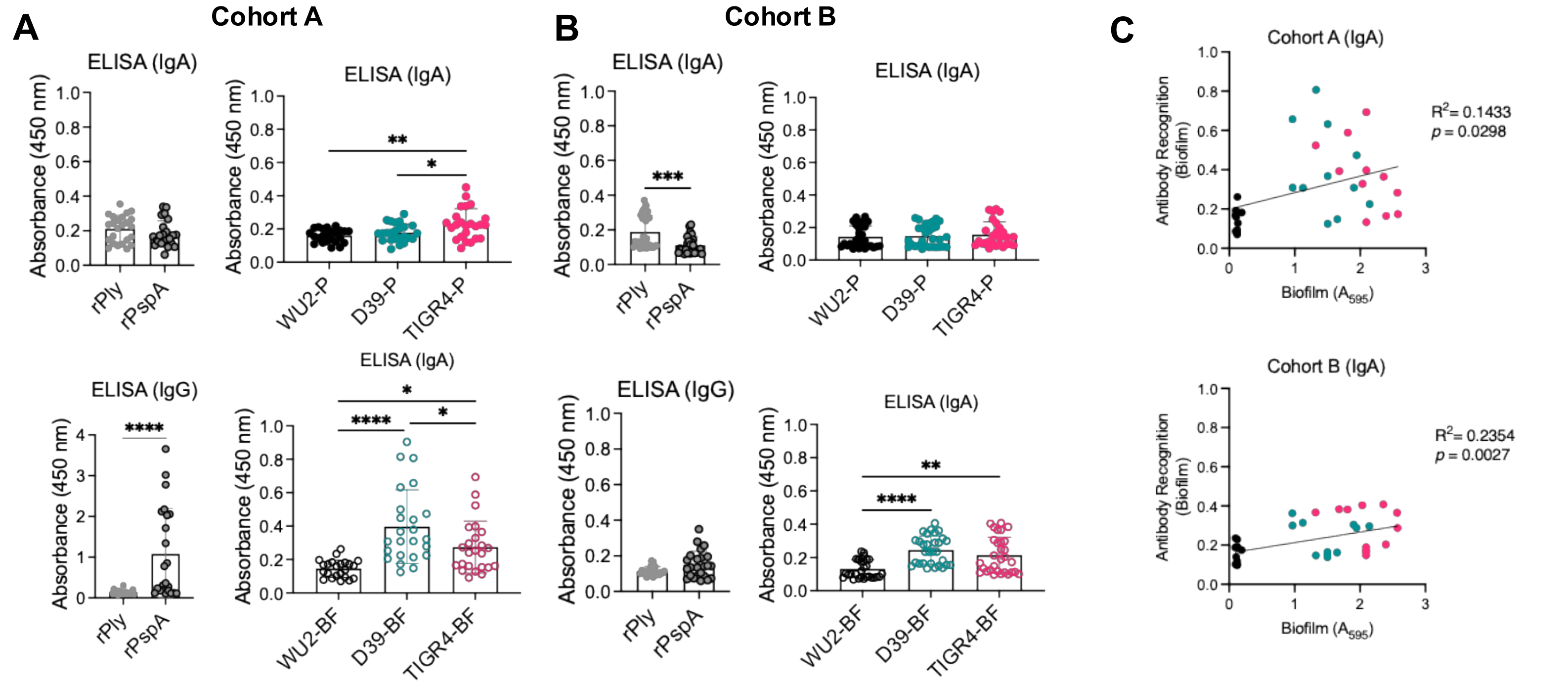

S7 Fig. Repeated murine *Spn* colonization elicits a strain-dependent humoral response associated with biofilm formation. Recombinant (r) protein and equal amounts of whole bacterial cell lysates grown planktonically (P) or in a biofilm (BF) from three *Spn* strains WU2 (serotype 3), D39 (serotype 2), and TIGR4 (serotype 4) were run on ELISAs and individually probed with serum (1:1000) from RAMPC$_3$ mice in **(A)** Cohort A and **(B)** Cohort B after the third colonization event. Secondary antibody α-mouse IgA and IgG (1:10000). Each dot is one mouse sample. N=24-30 over two separate experiments. **(C)** Linear regression correlation between ability of *Spn* strains to form biofilms and a ratio of IgA antibody recognition to biofilm antigens (strain-assigned colors corresponding to panel A). One-way ANOVA or Mann-Whitney t-test and mean with standard deviation. *=p≤0.0332; **=p≤ 0.002; ***=p ≤0.0002 ; ****=p≤ 0.0001.
