# Supplementary material for: Biofilm formation during pneumococcal carriage imprints naturally acquired humoral immunity
Source: PLoS Pathog. 2026 Jul 28;22(7):e1013826. doi: 10.1371/journal.ppat.1013826 (PMC13426961; doi:10.1371/journal.ppat.1013826)
Supplement: S8 Fig — (PDF) [file ppat.1013826.s008.pdf]

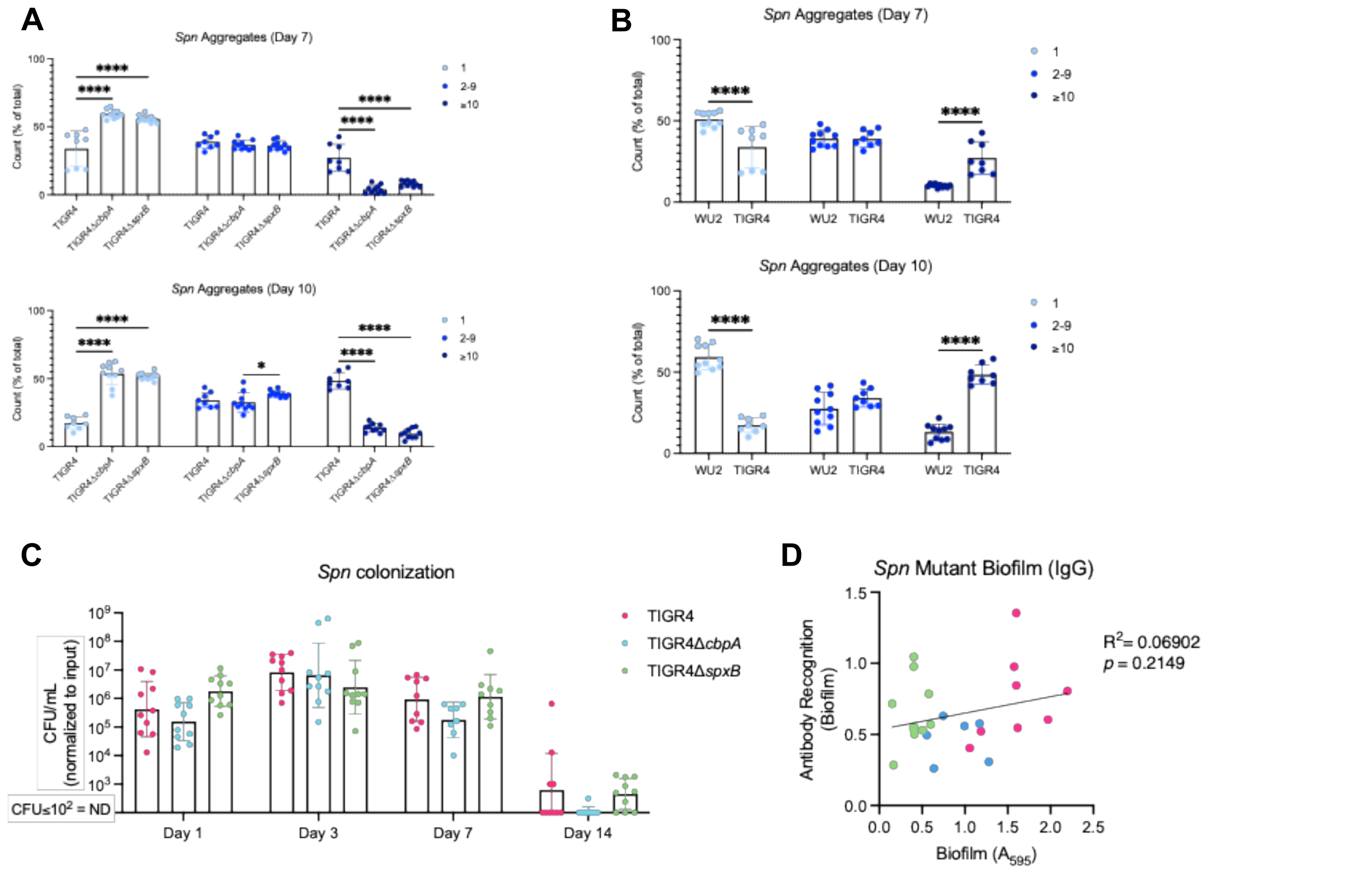

S8 Fig. In vivo biofilm aggregate formation is dependent on *Spn* strain. 9-week-old C57BL/6J female mice were intranasally inoculated with 10^4 CFU of **(A)** TIGR4 or biofilm-deficient mutants in the TIGR4 background (TIGR4Δ*cbpA* and TIGR4Δ*spxB*) and **(B)** WU2. After 7 or 10 days post-colonization, nasal washes in PBS were collected, stained with crystal violet, and observed under a microscope. The number of aggregates was counted and individual pneumococci analyzed as a percent of the total count (see methods). **(C)** Bacterial burden was determined over a 2-week period post-inoculation by colony forming units (CFUs) obtained from nasal washes with saline. Each dot is one mouse. N=8-10 per group over one experiment. **(D)** Linear regression correlation between ability of *Spn* biofilm-deficient mutant strains to form biofilms and a ratio of IgG antibody recognition to biofilm antigens (strain-assigned colors corresponding to panel C). Not detected (ND) ≤ 10^2 CFU/mL. Two-way ANOVA and mean with standard deviation or Median with 95% confidence interval (CI). ****=p≤ 0.0001.
