# Supplementary material for: Biofilm formation during pneumococcal carriage imprints naturally acquired humoral immunity
Source: PLoS Pathog. 2026 Jul 28;22(7):e1013826. doi: 10.1371/journal.ppat.1013826 (PMC13426961; doi:10.1371/journal.ppat.1013826)
Supplement: S9 Fig — (PDF) [file ppat.1013826.s009.pdf]

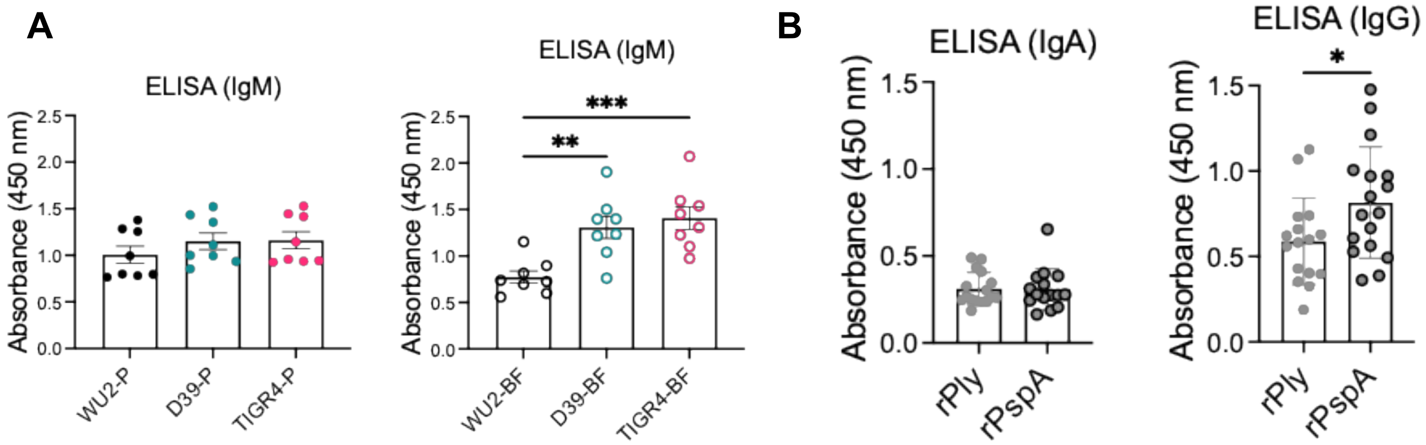

S9 Fig. Serum antibodies from asymptomatic colonized human adults recognize *Spn* antigens depending on strain. (A) Equal amounts of whole bacterial cell lysates grown planktonically (P) or in a biofilm (BF) from three *Spn* strains WU2 (serotype 3), D39 (serotype 2), and TIGR4 (serotype 4) were run on ELISAs and probed using serum (1:1000) from asymptomatically colonized adults (aged 40-82). Secondary antibody α-human IgM (1:10000). Each dot is one human sample. N=8 over one experiment. One-way ANOVA with standard deviation is shown. **(B)** Recombinant (r) protein from pneumolysin (rPly) and pneumococcal surface protein A (rPspA) were run on ELISAs and individually probed with serum (1:1000) from asymptomatically colonized adults (aged 40-82) and secondary antibody α-human IgA and IgG (1:10000). Each dot is one human sample. N=17 over one experiment. Mann-Whitney t-test and mean with standard deviation. *=$p \leq 0.0332$; **=$p \leq 0.002$; ***=$p \leq 0.0002$.
