# Supplementary material for: Biofilm formation during pneumococcal carriage imprints naturally acquired humoral immunity
Source: PLoS Pathog. 2026 Jul 28;22(7):e1013826. doi: 10.1371/journal.ppat.1013826 (PMC13426961; doi:10.1371/journal.ppat.1013826)
Supplement: S10 Fig — (PDF) [file ppat.1013826.s010.pdf]

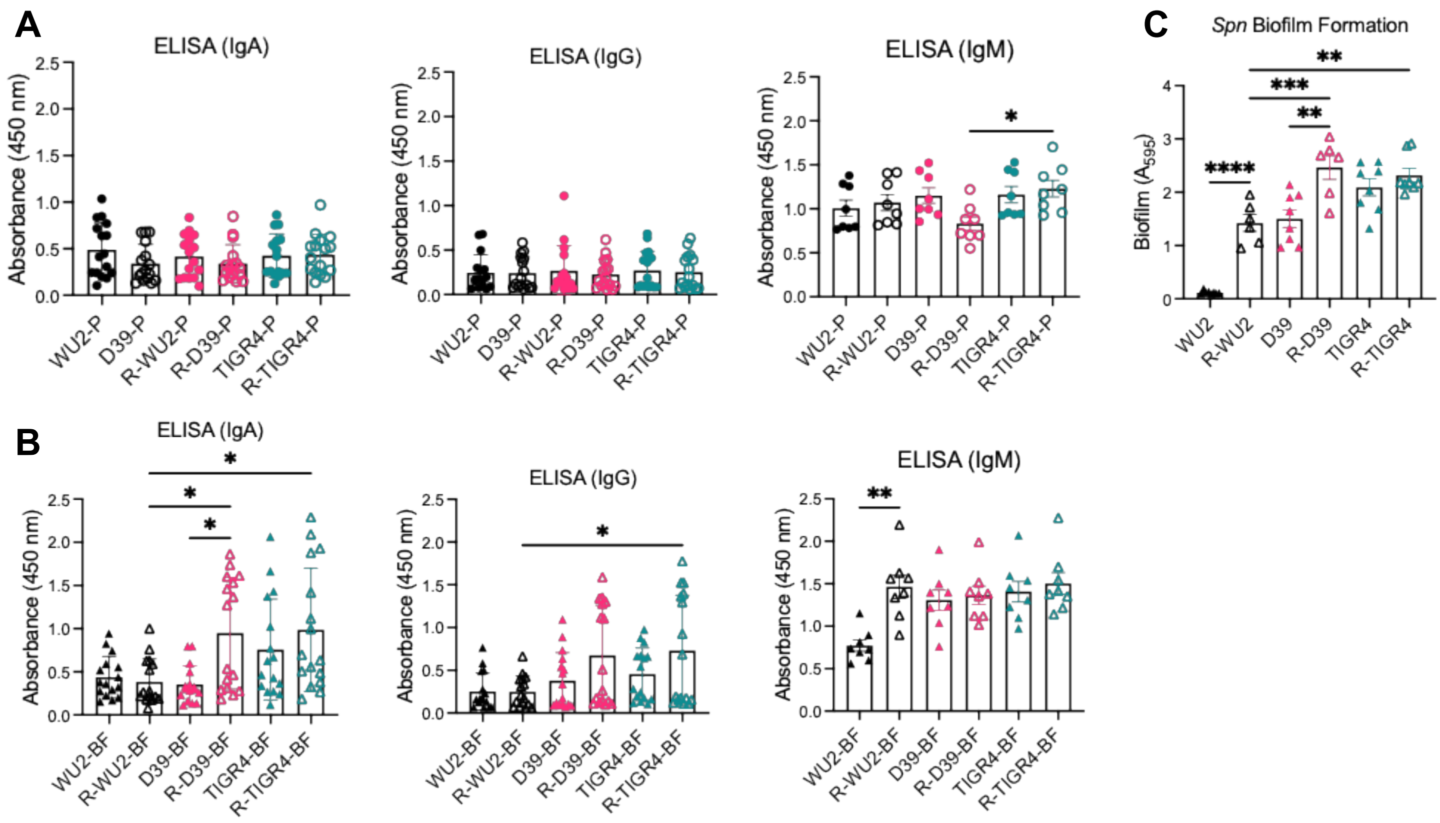

S10 Fig. *Spn* antigen immunoglobulin specificity in asymptomatic adult carriers is not capsule-dependent. Equal amounts of whole cell lysate (WCL) from the *Spn* strains WU2 (serotype 3), D39 (serotype 2), and TIGR4 (serotype 4) grown **(A)** planktonically (P) and as a **(B)** biofilm (BF) and unencapsulated (R) and capsulated versions were run on ELISAs and individually probed with serum (1:1000) from asymptomatically colonized adults (aged 40-82) and secondary α-human IgA, IgG, and IgM (1:10000). Each dot is one human sample. N=8-17 over one experiment. **(C)** Biofilm formation for *Spn* unencapsulated and capsulated strains as determined by crystal violet assay. N=6-8. One-way ANOVA and mean with standard deviation. *=p≤0.0332; **=p≤ 0.002; ***=p ≤0.0002; ****=p≤ 0.0001.
