# Supplementary material for: Biofilm formation during pneumococcal carriage imprints naturally acquired humoral immunity
Source: PLoS Pathog. 2026 Jul 28;22(7):e1013826. doi: 10.1371/journal.ppat.1013826 (PMC13426961; doi:10.1371/journal.ppat.1013826)
Supplement: S11 Fig — (PDF) [file ppat.1013826.s011.pdf]

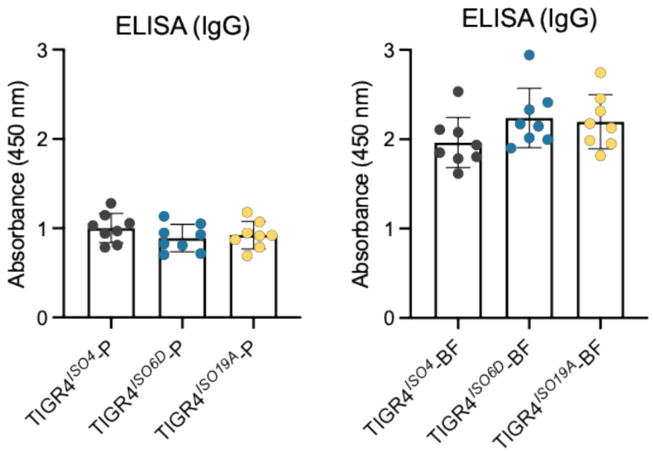

S11 Fig. Serum antibodies from asymptomatic colonized human adults recognize *Spn* antigens regardless of capsule type in the same genetic background. Equal amounts of whole bacterial cell lysates grown planktonically (P) or in a biofilm (BF) from three TIGR4 isogenic capsule switch mutant *Spn* strains (isotype 4, 6D, and 19A) were run on ELISAs and individually probed with serum (1:1000) from asymptomatically colonized adults (aged 40-82) and secondary α-human IgG (1:10000). Each dot is one human sample. N=8 over one experiment. Mean with standard deviation shown.
