# Supplementary material for: Biofilm formation during pneumococcal carriage imprints naturally acquired humoral immunity
Source: PLoS Pathog. 2026 Jul 28;22(7):e1013826. doi: 10.1371/journal.ppat.1013826 (PMC13426961; doi:10.1371/journal.ppat.1013826)
Supplement: S12 Fig — (PDF) [file ppat.1013826.s012.pdf]

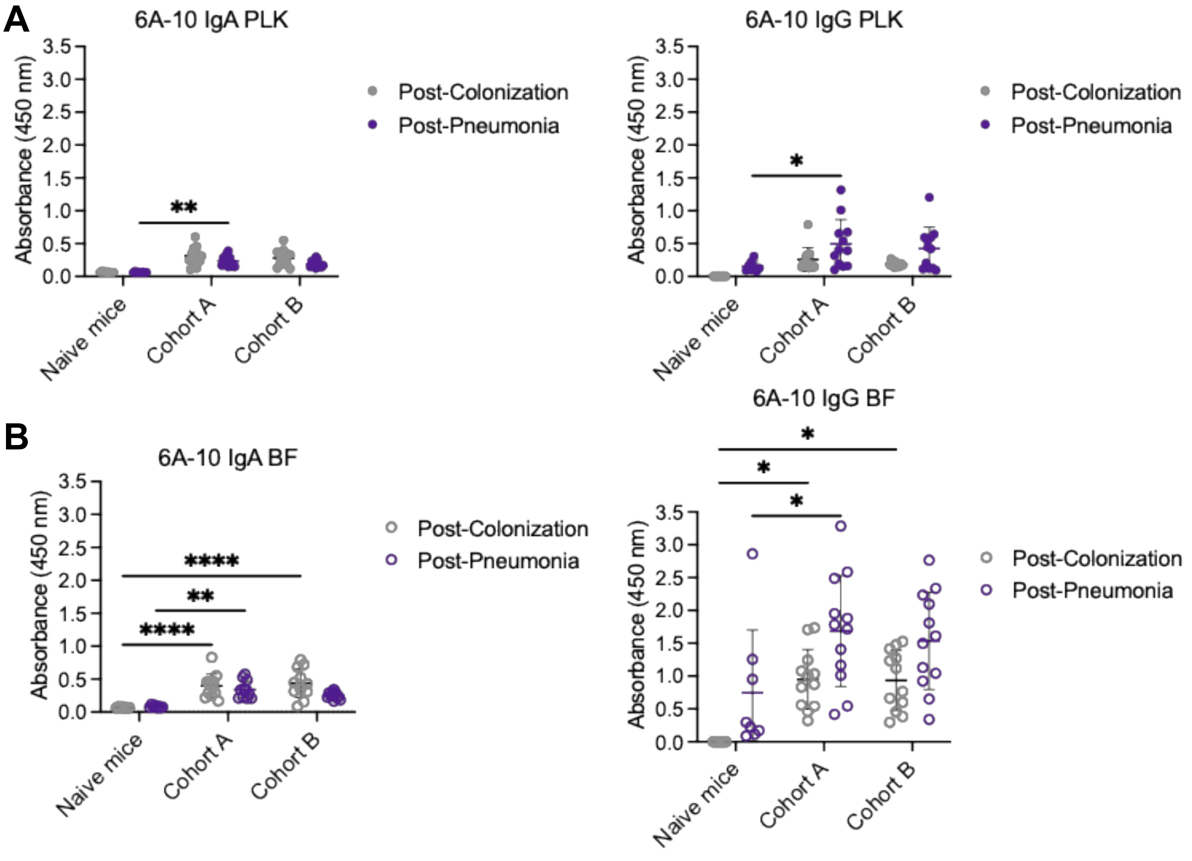

S12 Fig. Repeated asymptomatic colonization with *Spn* partially protects against pneumococcal pneumonia. A cohort of age-matched female naïve mice and the $RAMPC_3$ mice from both Cohorts A and B were intratracheally challenged with 10^4 CFU of *Spn* strain 6A-10 (serotype 6A) (see methods). Equal amounts of whole bacterial cell lysates grown planktonically (PLK) or in a biofilm (BF) from 6A-10 were run on ELISAs and individually probed with mouse sera (1:1000) from surviving mice and secondary α-mouse IgA or IgG (1:10000). Each dot is one mouse sample. N=8-12 per group over two separate experiments. Two-way ANOVA and mean with standard deviation. *=p≤0.0332; **=p≤ 0.002; ****=p≤ 0.0001.
