# Supplementary material for: Biofilm formation during pneumococcal carriage imprints naturally acquired humoral immunity
Source: PLoS Pathog. 2026 Jul 28;22(7):e1013826. doi: 10.1371/journal.ppat.1013826 (PMC13426961; doi:10.1371/journal.ppat.1013826)
Supplement: S13 Fig — (PDF) [file ppat.1013826.s013.pdf]

A

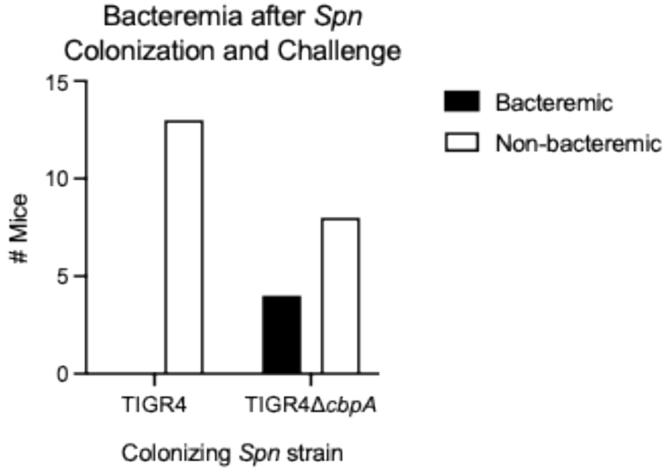

	Bacteremic	Non-Bacteremic
TIGR4	0	13
TIGR4Δ*cbpA*	4	8
Total	4	21
Chi^2 p-value	0.0231	

B

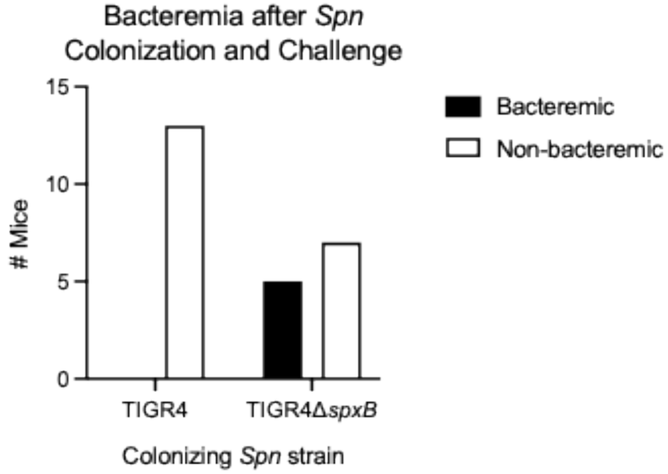

	Bacteremic	Non-Bacteremic
TIGR4	0	13
TIGR4Δ*spxB*	5	7
Total	5	20
Chi^2 p-value	0.0093	

S13 Fig. *Spn* deficient in biofilm formation are less protected against pneumococcal pneumonia following colonization. 9-week-old C57BL/6J female mice were intranasally inoculated with 10^4 CFU of **(A)** TIGR4, TIGR4Δ*cbpA* or **(B)** TIGR4Δ*spxB.* After one month, mice were intratracheally challenged with 10^3 CFU of a different *Spn* strain 6A-10 (serotype 6A). Bacterial burden in the blood was recorded, and Chi^2 analysis was used to compare bacteremic versus non-bacteremic mice (tables). N=12-13 per group over one experiment.
