# Supplementary material for: Biofilm formation during pneumococcal carriage imprints naturally acquired humoral immunity
Source: PLoS Pathog. 2026 Jul 28;22(7):e1013826. doi: 10.1371/journal.ppat.1013826 (PMC13426961; doi:10.1371/journal.ppat.1013826)
Supplement: S14 Fig — (PDF) [file ppat.1013826.s014.pdf]

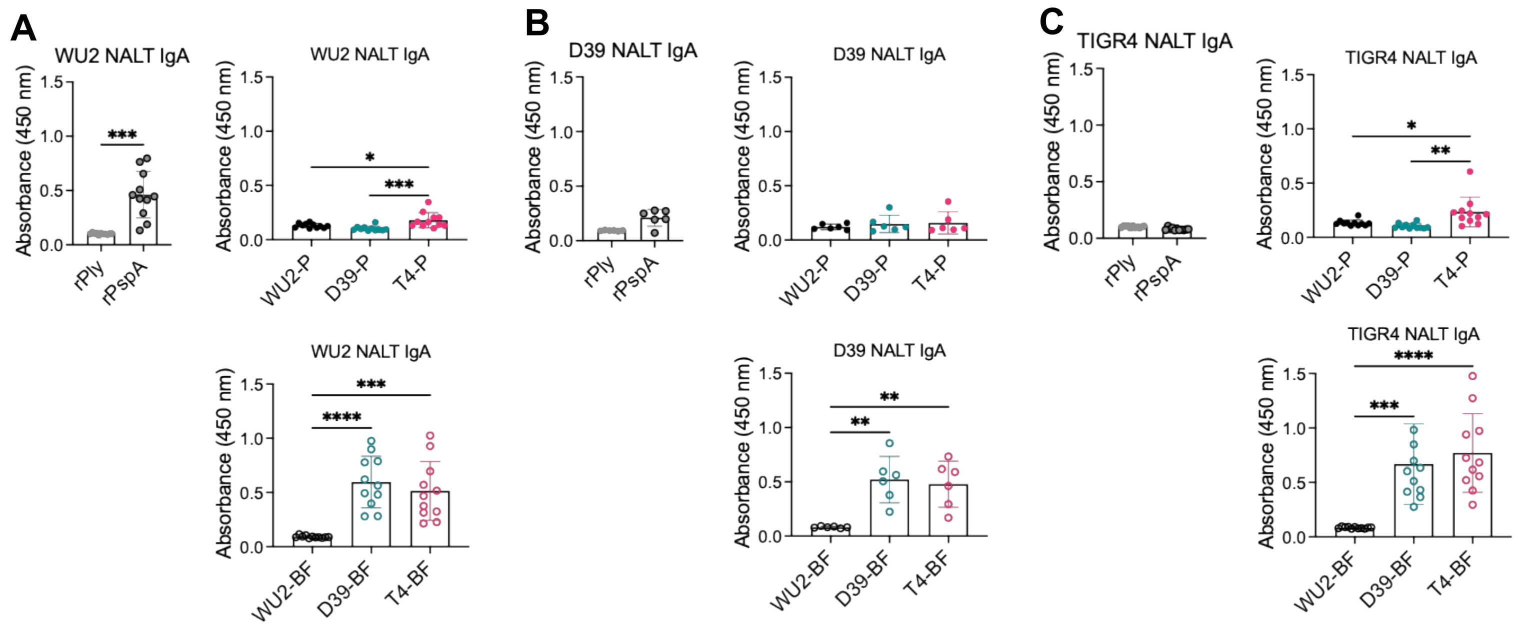

S14 Fig. Serum IgA reactivity is comparable to mucosal tissue-derived antibody responses. 9-week-old C57BL/6J male and female mice were inoculated intranasally with *Spn* strains **(A)** WU2 (serotype 3), **(B)** D39 (serotype 2), and **(C)** TIGR4 (serotype 4) (10^4 CFU) as single colonization events. After 2 weeks, nasal associated lymphoid tissue (NALT) was harvested (see methods), homogenized in 1 mL of saline, and used at (1:10) as primary antibody in ELISA analysis against recombinant (r) protein and equal amounts of whole bacterial cell lysates grown planktonically (P) or in a biofilm (BF) from WU2, D39, and TIGR4. Secondary mouse α-mouse IgA was used at (1:10000). Each dot is one mouse sample. N=6-11 over one experiment. One-way ANOVA and mean with standard deviation. *=p≤0.0332; **=p≤ 0.002; ***=p ≤0.0002; ****=p≤ 0.0001.
