# Supplementary material for: Biofilm formation during pneumococcal carriage imprints naturally acquired humoral immunity
Source: PLoS Pathog. 2026 Jul 28;22(7):e1013826. doi: 10.1371/journal.ppat.1013826 (PMC13426961; doi:10.1371/journal.ppat.1013826)

Figure 2 uncropped blots

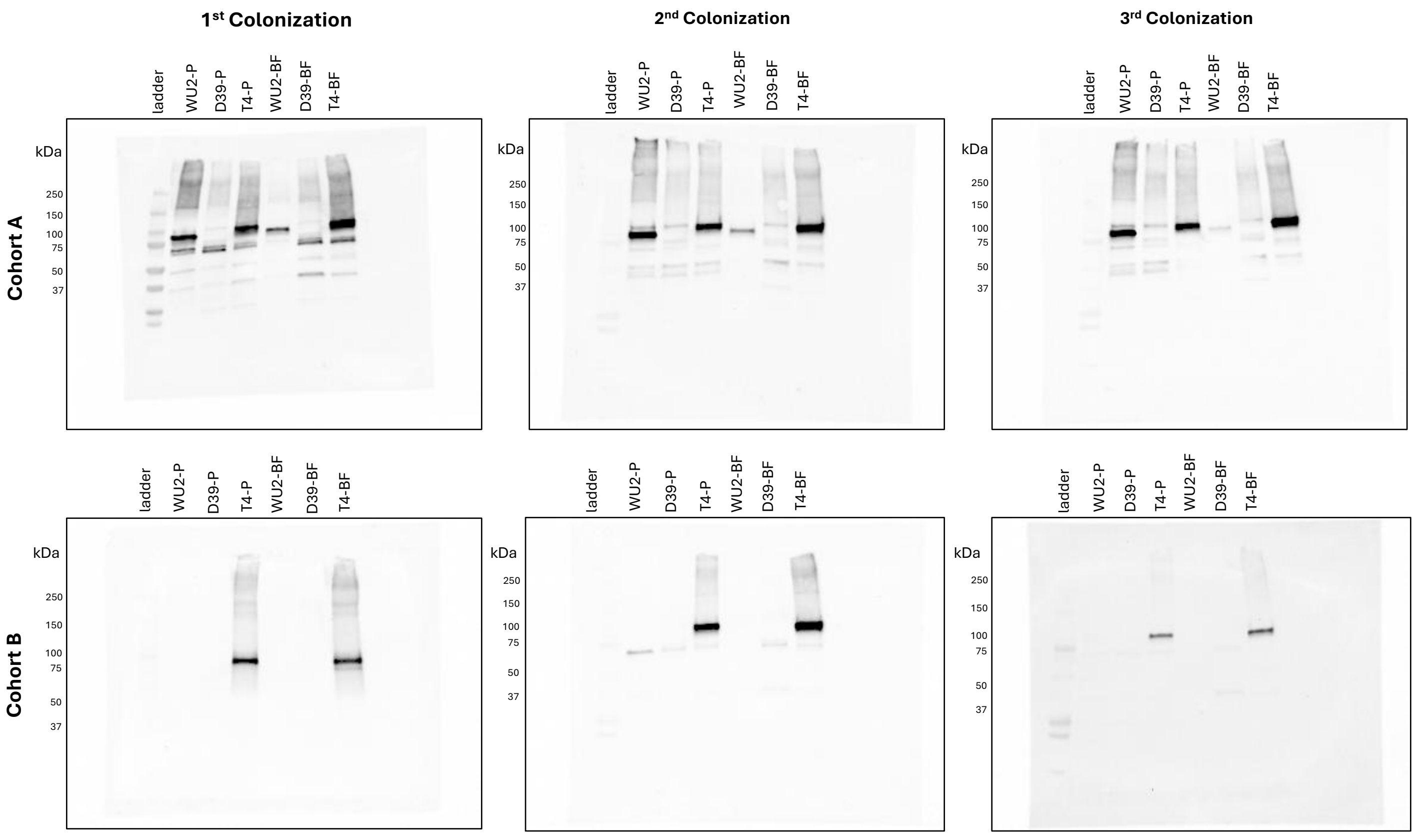

Supplemental Figure 2 uncropped gel and blots

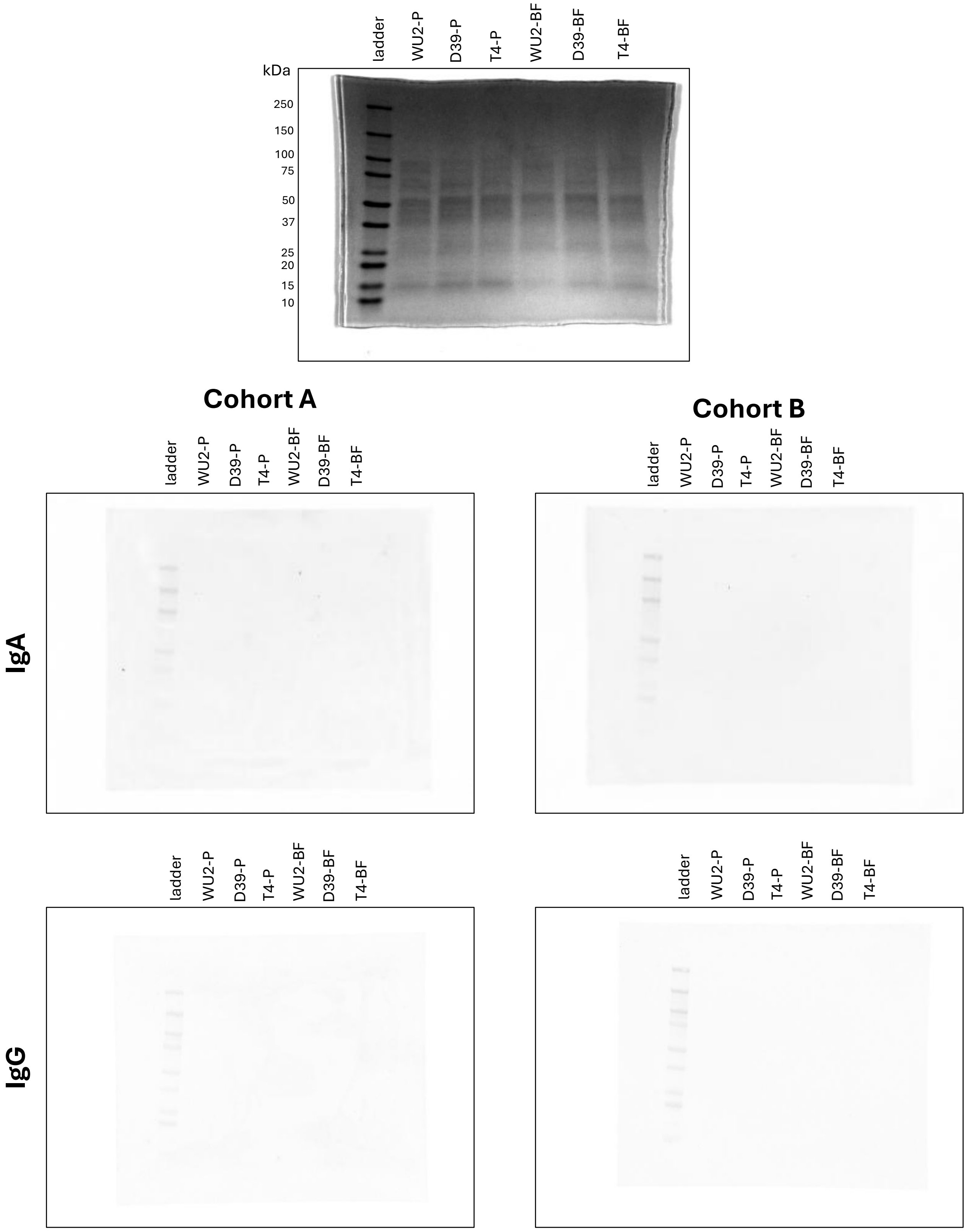

Supplemental Figure 3 uncropped blots

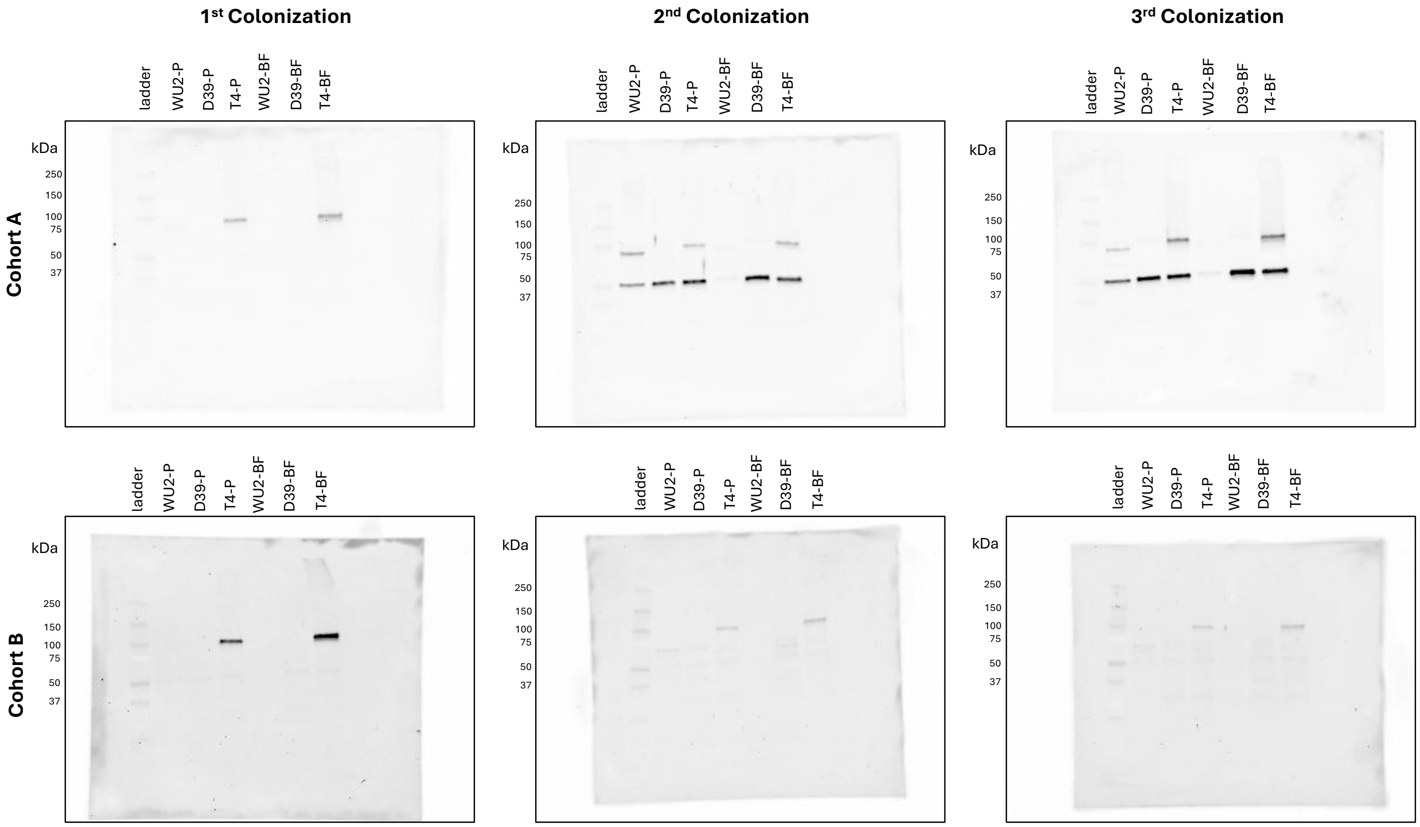

Supplement: S1 Raw Image — (PDF) [file ppat.1013826.s015.pdf]
