# Supplementary material for: Biofilm formation during pneumococcal carriage imprints naturally acquired humoral immunity
Source: PLoS Pathog. 2026 Jul 28;22(7):e1013826. doi: 10.1371/journal.ppat.1013826 (PMC13426961; doi:10.1371/journal.ppat.1013826)
Supplement: S1 Table — See methods and references for details. (DOCX) [file ppat.1013826.s016.docx]

**Supplemental Table 1. Bacterial strains used.**

| **Strain** | **Serotype** | **Global Pneumococcal Sequencing Cluster (GPSC)** | **Genotype** | **Reference** |
| --- | --- | --- | --- | --- |
| D39 | 2 | 622 | Clinical Isolate | Lanie et al. 2007 |
| R6 | 2 | 622 | Unencapsulated spontaneous mutant | UAB (Carlos Orihuela) |
| CI-SPN2 | 2 |  | Clinical Isolate-Invasive | UAB (David Briles) |
| WU2 | 3 | 83 | Clinical Isolate | Hollingshead, Becker et al. 2000 |
| RWU2 | 3 | 83 | Δwhole_cps::cat | UAB (Carlos Orihuela) |
| CI-SPN3(C) | 3 |  | Clinical Isolate-Carriage | Universidad de la Sabana (Luis F. Reyes) |
| CI-SPN3(I) | 3 |  | Clinical Isolate-Invasive | Universidad de la Sabana (Luis F. Reyes) |
| TIGR4 | 4 | 27 | Clinical Isolate | Tettelin et al. 2001 |
| RTIGR4 | 4 | 27 | Δwhole_cps::cat | UAB (Carlos Orihuela) |
| CI-SPN4 | 4 |  | Clinical Isolate | Universidad de la Sabana (Luis F. Reyes) |
| 6A-10 | 6A | 64 | Clinical Isolate |  |
| R6A-10 | 6A | 64 | Δwhole_cps::cat | UAB (Carlos Orihuela) |
| TIGR4∆*cbpA* | 4 | 27 | Δ*cbpA*::*aadA* | Brissac et al. 2018 |
| TIGR4∆*spxB* | 4 | 27 | Δ*spxB*::*erm* | Brissac et al. 2018 |
| TIGR4*^ISO4^* | 4 | 27 | ∆cps::cps4 | UAB (Carlos Orihuela) |
| TIGR4*^ISO6D^* | 6D | 27 | ∆cps::cps6D | UAB (Carlos Orihuela) |
| TIGR4*^ISO19A^* | 19A | 27 | ∆cps::cps19A | UAB (Carlos Orihuela) |
