# Supplementary material for: Biofilm formation during pneumococcal carriage imprints naturally acquired humoral immunity
Source: PLoS Pathog. 2026 Jul 28;22(7):e1013826. doi: 10.1371/journal.ppat.1013826 (PMC13426961; doi:10.1371/journal.ppat.1013826)
Supplement: S3 Table — See reference Olivella-Gomez et al. 2025. (DOCX) [file ppat.1013826.s018.docx]

**Supplemental Table 3. Patient cohort information from human individuals naturally colonized with *Spn.***

| Patient | Age (years) | Sex | Vaccinated at baseline | Comorbidity | Smoker |
| --- | --- | --- | --- | --- | --- |
| 1 | 40 | F | N | Y | Y |
| 2 | 42 | F | N | Y | N |
| 3 | 42 | M | N | Y | N |
| 4 | 61 | M | N | Y | N |
| 5 | 62 | M | N | Y | N |
| 6 | 63 | F | N | Y | Y |
| 7 | 63 | F | N | Y | N |
| 8 | 65 | F | N | Y | N |
| 9 | 68 | M | Y | Y | N |
| 10 | 69 | F | N | Y | N |
| 11 | 69 | M | N | Y | N |
| 12 | 69 | F | N | Y | Y |
| 13 | 72 | F | N | Y | Y |
| 14 | 72 | M | N | Y | N |
| 15 | 73 | M | Y | Y | N |
| 16 | 73 | F | N | Y | N |
| 17 | 82 | F | N | Y | N |
|  | Average: 64 | F:M::10:7 | Frequency: 12% | Hypertension: 53%  CKD: 47%  Diabetes: 29% | Frequency: 24% |

**Key:**

F= Female

M= Male

Y= Yes

N= No

CKD= chronic kidney disease
